# Simple Parameters and Data Processing for Better Signal-to-Noise and Temporal Resolution in *In Situ* 1D NMR Reaction Monitoring

**DOI:** 10.1021/acs.joc.4c01882

**Published:** 2024-11-01

**Authors:** Annabel Flook, Guy C. Lloyd-Jones

**Affiliations:** School of Chemistry, University of Edinburgh, Joseph Black Building, Edinburgh EH9 3FJ, United Kingdom

## Abstract

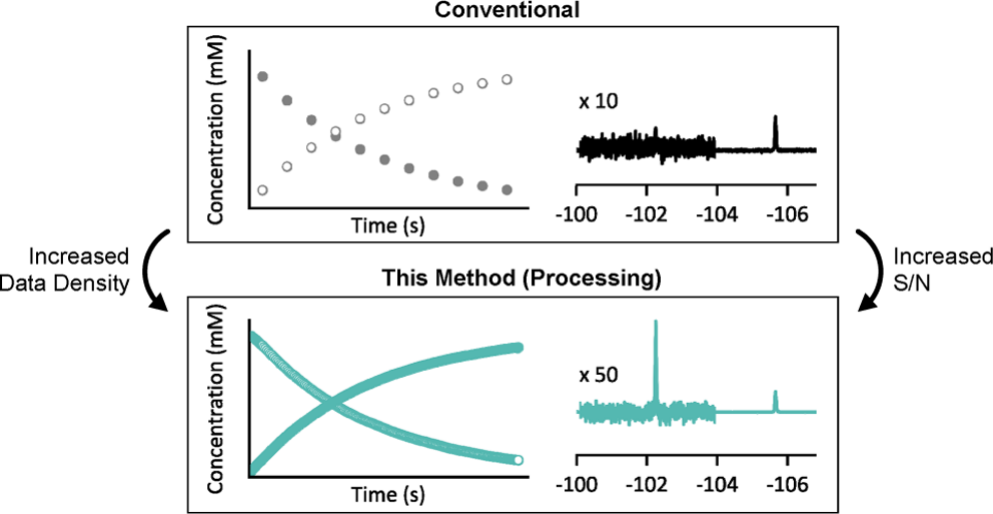

*In situ* 1D NMR spectroscopic reaction monitoring allows detailed investigation of chemical kinetics and mechanism. Concentration versus time data are derived from a time series of NMR spectra. Each spectrum in the series is obtained by Fourier transform of the corresponding FID. When the spectrometer outputs FIDs recorded from multiple scans, the spectra benefit from an increase in signal-to-noise (S/N). However, this reduces the number of FIDs and, thus, kinetic data points. We report a simple alternative in which the same number of scans is acquired by the spectrometer, but each scan is saved independently. Signal averaging is then conducted by postacquisition processing. This leads to an increase in both the S/N and the number of kinetic data points and can avoid “overaveraging” effects. The entire series of single-scan FIDs spanning the reaction lifetime can be summed to yield a “total reaction spectrum” in which intermediates can be identified. The method can be applied in coherence with phase cycling to minimize spectral distortion during solvent signal suppression. Overall, the approach simplifies the preacquisition parameters to the estimation of the reaction duration and *T*_1_^max^ and then the selection of the pulse angle, θ, and scan repetition time, τ_R_, without the need to set the signal averaging before the experiment.

## 1 Introduction

### 1.1 Reaction Monitoring Data

*In situ* NMR spectroscopic reaction monitoring can provide unique insight into kinetics, identities of intermediates, and reaction mechanisms in organic and organometallic chemistry.^1^ Using this technique, the reaction is initiated in an NMR tube, or flow system, and then monitored continuously in the probe of a high-field or benchtop spectrometer.^2^ A series of NMR spectra are collected over the lifetime of the reaction, typically from minutes to a few hours.

The inherently low sensitivity of NMR compared to other spectroscopic techniques means that the conditions and acquisition parameters must be carefully selected for the resulting spectra to provide suitable temporal concentration data. While graphical methods^3^ can be applied to qualitatively interpret relatively sparse datasets from simple processes, this is not the case for quantitative techniques,^4^ including analytical rate equations, steady state approximations, and numerical methods simulations.^1a^ Thus, the determination of rate constants, linear free energy relationships, secondary and heavy atom kinetic isotope effects (KIEs),^4b^ partitioning coefficients,^4c^ kinetic resolution factors,^4d^ entrainment,^4e^ and numerous other mechanistic probes, requires substantial datasets. In the case of *in situ* NMR spectroscopic reaction monitoring, each of the individual NMR spectra must therefore be acquired with sufficient signal-to-noise (S/N) to resolve and quantify species across the full range of concentrations present in the reaction at that specific time-point. Conversely, there must be a sufficient number and distribution of kinetic data points acquired over the reaction lifetime to reliably characterize the full process.

Conventional adjustment of parameters to improve one aspect (S/N versus temporal resolution of the kinetic data) results in the detriment of the other, and this dichotomy is a pervasive issue in reaction monitoring by NMR spectroscopy.^1a,1b^ A variety of methods to enhance S/N,^5^ including hardware (e.g., higher field magnets, cryoprobes) and techniques (e.g., hyperpolarization)^6^ are available. However, these require specialist equipment to implement, making them less appealing for general purpose reaction monitoring, particularly when wet chemistry is conducted near to,^2^ or within,^7^ the spectrometer. Postacquisition processing methods such as window functions^8^ and neural networks,^9^ typically provide only minor enhancements to S/N, or are not readily implemented by the nonspecialist.

More elementary approaches, such as increasing reactant concentrations (greater S/N) or reducing the reaction temperature (greater temporal resolution), as well as more extensive modifications such as changing stoichiometries, catalyst loadings, or solvent, can also be effective. However, great care must be taken in the interpretation of the resulting data: too extensive a change in conditions can impact the kinetic and mechanistic features, and the system may no longer represent the original process of interest.^1a^

## 2 Results and Discussion

Herein, we report on the systematic selection of pulse angle and scan interval, with postacquisition processing of combinations of single-scan free induction decays (FIDs). Correctly applied, the method increases both the S/N and the number of kinetic data points, without requiring additional spectrometer time.

To contextualize these advantages, we first briefly review some of the pertinent features for conventional reaction monitoring by *in situ* NMR spectroscopy.

### 2.1 Quantitative NMR

A prerequisite for *in situ* NMR reaction monitoring is the extraction of accurate temporal concentration data for all species of interest in the NMR spectra. The signal intensities of all the observed nuclei must therefore be directly proportional to their concentration. To maintain this quantitative NMR condition,^10^ throughout the reaction, the magnetization (*M*_τ_) must be allowed to approach full recovery between scans, *M*_τ_/*M*_0_ ≈ 1. Prior estimation of the longitudinal relaxation time^11^ (*T*_1_) for each of the nuclei being monitored is thus essential.

The pulse angle (θ) determines the fractional intensity (*f*_max_; from 0–1) of the NMR signals, eq 1. The slowest relaxing nucleus (*T*_1_^max^) and the pulse angle θ, then collectively dictate the minimum interval required between consecutive scans. A commonly accepted threshold for this condition when θ = 90° (*f*_max_ = 1) is setting the scan repetition time, τ_R_,^12^ to be ≥5 × *T*_1_^max^ which ensures *M*_τ_/*M*_0_ ≥ 0.993 for all the monitored nuclei, eq 2.^13^
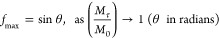
1
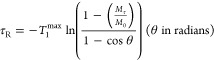
2
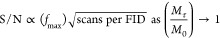
3

### 2.2 Signal Averaging

**Figure 1:**
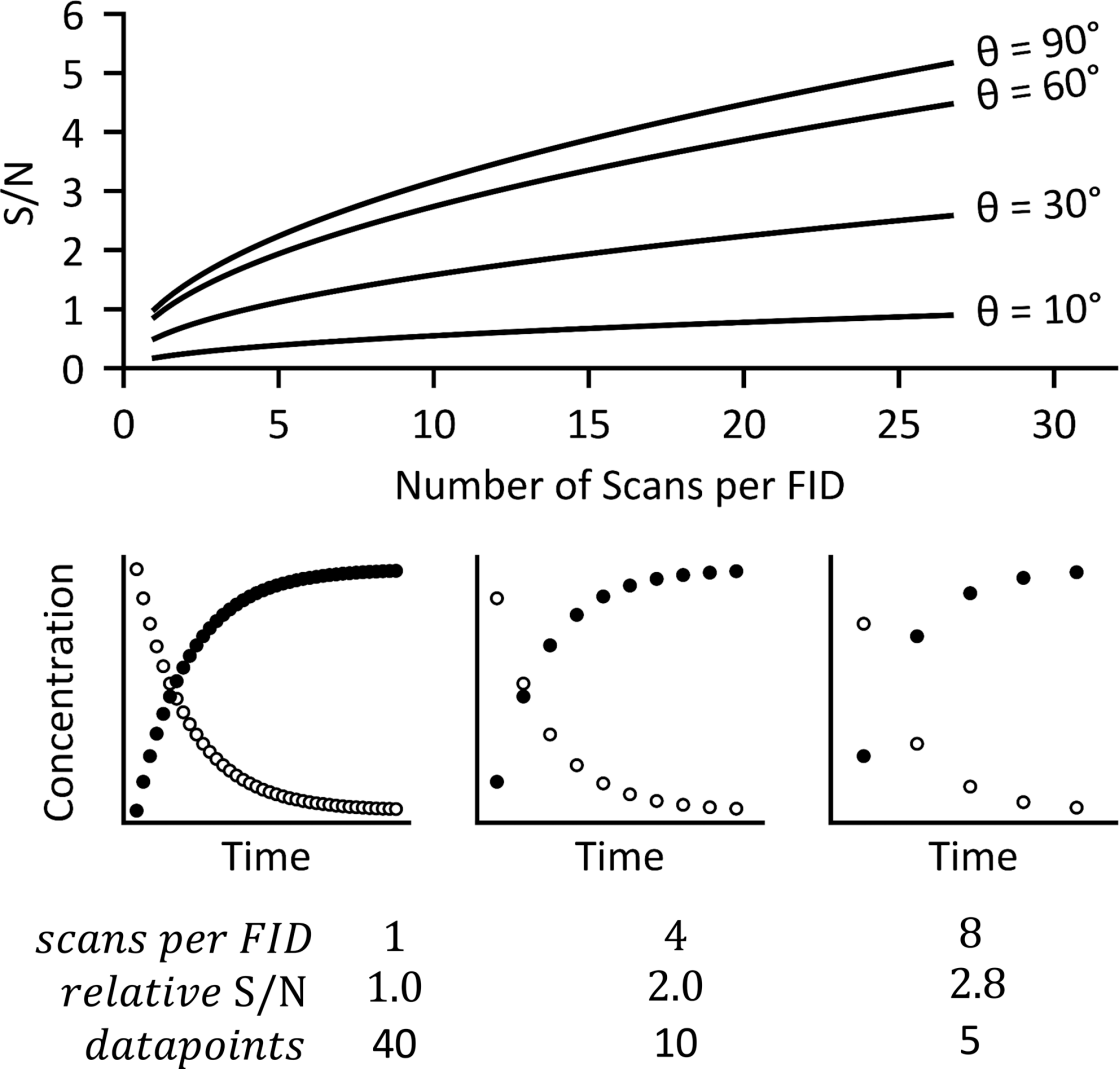
Upper: relationships between signal-to-noise (S/N), the pulse angle, θ, and the number of scans averaged per FID, with repetition time, τ_R_, set to restore equilibrium magnetization, *M*τ/*M*_0_ ≈1, between scans. Lower: illustration of the compromise between the S/N and number of kinetic data points when signal averaging (scans per FID) in conventional monitoring, θ = 90°.

In routine 1D NMR spectroscopy, the raw data from a series of scans are accumulated in the spectrometer software and then saved as a single FID for processing. The resulting gain in S/N in the signal-averaged NMR spectrum, eq 3, is determined by the number of scans accumulated, with this value being set prior to the experiment.^5b,14,15^ When applied conventionally to reaction monitoring, the extent of signal averaging predictates the number of kinetic data points that are generated during a defined reaction lifetime, and their temporal spacing, Figure 1.

When reaction monitoring using conventional averaging, the scan repetition time, τ_R_, can be reduced by decreasing the pulse angle, θ, eq 2. Since scans can then then repeated over a shorter time-period, the temporal spacing is reduced and the number of the kinetic data points increased. For example, using θ = 30° allows *N* to be increased by a factor of 1.7 compared to when θ = 90°, albeit at the cost of S/N (*f*_max_ = 0.5).^13,14^ However, as previously noted,^15^ and further discussed below, reducing the pulse angle, θ, from 90° is not always the best approach for reaction monitoring.

### 2.3 Single-Scan FID Data Series

The generation of a single FID by transiently accumulating the raw data from multiple scans is a legacy from the early days of pulse NMR, when data storage and computational power were many orders of magnitude less advanced than now.^16^ Without this limitation, a large series of FIDs can now be acquired and stored independently, each arising from a single scan collected at intervals of τ_R_ set to maintain the quantitative condition.^10^

While single-scan NMR spectra can be employed for *in situ* NMR reaction monitoring,^1^ for example via a “pseudo 2D-mode kinetics experiment”,^17^ this loses the beneficial effect of signal averaging, eq 3 and Figure 1. Below we show that signal averaging can still be harnessed via postacquisition processing using a moving average of *n-*scans,^18^ (*n* > 1) that is propagated through the single-scan FID data-stack. This allows postexperiment variations in the extent of scan averaging to optimize the number of kinetic data points and S/N, while avoiding artifacts from overaveraging.

The entire series of FIDs spanning the reaction lifetime can also be summed to yield, after Fourier transform, a single “total reaction spectrum” in which the maximum S/N is attained. This spectrum provides auxiliary information regarding the presence and chemical shift of low concentration side-products, or transient species such as intermediates. Strategic selection of values for *n* can also make best use of phase-cycling sequences to minimize spectral distortions, while still retaining temporal resolution in the kinetic data. Below we outline the method of application and then, using four simple examples, the utility of the approach.

### 2.4 Parameterization and Processing

**Figure 2:**
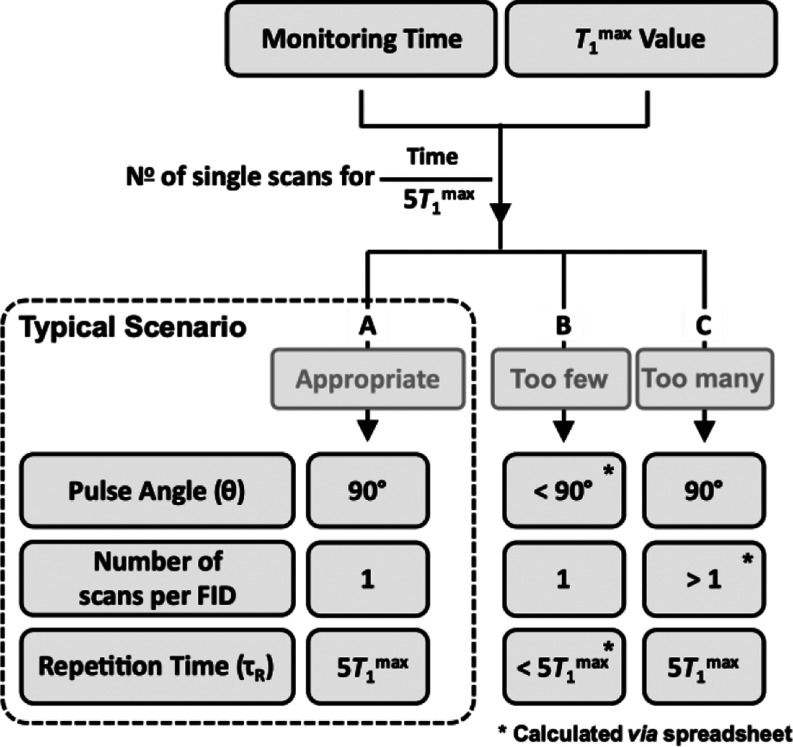
Parameter selection process for reaction monitoring using postacquisition signal averaging. See the Supporting Information for a spreadsheet that on input of reaction monitoring time and *T*_1_^max^, evaluates the pathway (A, B, C), the θ, scans per FID accumulated on the spectrometer, and τ_R_ values, visualizes the data density, S/N, and the effects of (over)averaging, and calculates the midtime points (*t*) to use with the resulting kinetic data.

Using the postacquisition approach, the process of pre-experiment parameter selection is simplified to that shown in Figure 2. After estimating the required reaction monitoring time and *T*_1_^max^,^11^ the Pathway (A, B, C) and values for θ and τ_R_, as defined by eqs 1 to 3, are calculated: see the Supporting Information for a preconfigured spreadsheet.

In most cases, the process will identify use of a 90° pulse (*f*_max_ = 1) with τ_R_ = 5 × *T*_1_, see Pathway A. For faster reactions, the repetition time, τ_R_, is reduced to allow generation of a sufficient number of kinetic data points, via a reduction in the pulse angle,^15^ θ, albeit at the cost of S/N (*f*_max_ < 1), Pathway B, see the Supporting Information Section S8 for discussion. Since each scan is saved separately, relatively slow reactions result in unnecessarily large datasets and lengthy processing. In such cases, some preliminary on-spectrometer FID averaging of multiple scans, Pathway C, is identified to be more time-efficient.

Irrespective of the pathway (A, B, or C) the postacquisition processing is carried out identically, with signal averaging applied to the scans in direct analogy to that conventionally employed during acquisition. The process can be readily automated via standard scripting methods, see the Supporting Information Section S11. The scans can be summed in blocks of any size (*n*) without overwriting the original data, and the resulting FIDs transformed into NMR spectra for normal processing techniques, such as phase and baseline correction. Integration of the species of interest, with reference to an internal standard, yields their concentration at time, *t*, set to be the midpoint in the block of *n* scans; see the spreadsheet in the Supporting Information for calculation of *t*. Only the original set of scans, i.e., neither the NMR spectra (frequency-domain data) nor the temporal concentration data, should be used for signal averaging as some of the processing steps from FID to spectrum are algorithmic and artifacts will be amplified, potentially with a loss in quantitation.

### 2.5 Comparison with Conventional Averaging

**Figure 3:**
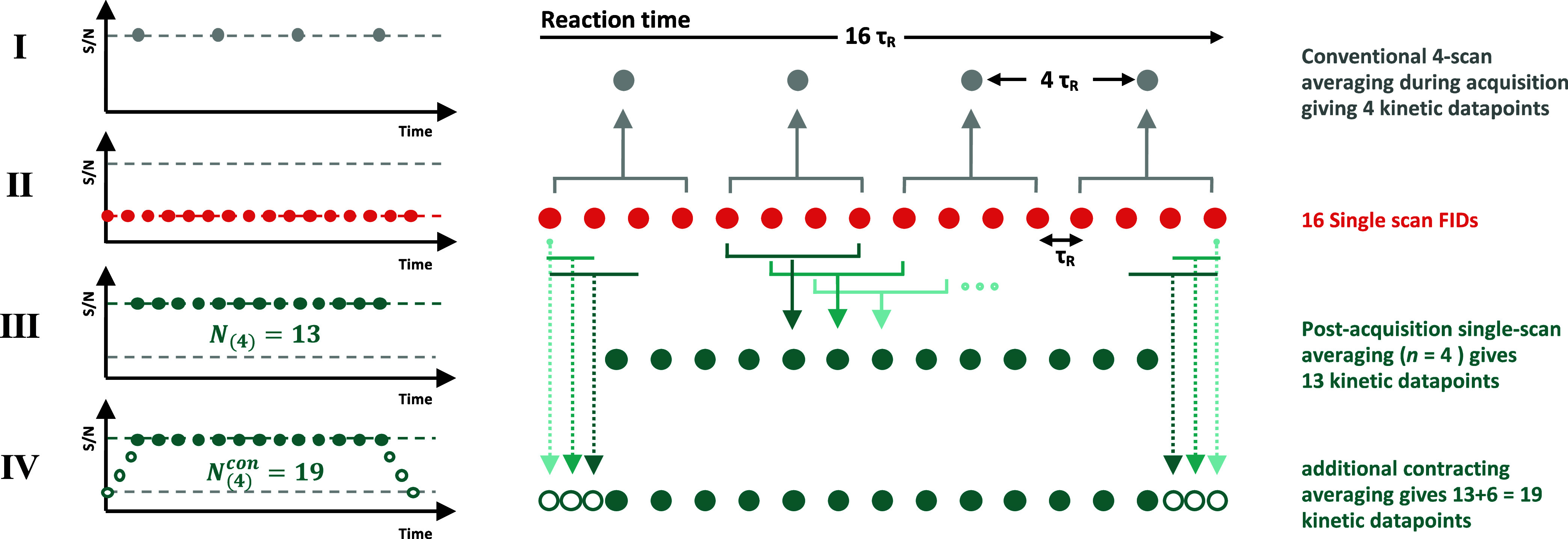
Schematic illustrations of number of kinetic data points arising from (I) conventional on-spectrometer signal averaging of 4 scans per FID and (II) single scan FID NMR spectra, i.e., with no averaging, over a reaction time of 16 τ_R_. Postacquisition signal averaging (III) of the single-scan data, using the same 4-scan window as the conventional method, and with additional contracting averaging applied at the termini (IV) leads to an increase in the number of kinetic data points (*N*_(4)_^con^ = 19) and the signal/noise (S/N).

Figure 3 illustrates the differences between conventional on-spectrometer averaging (**I**), single-scan data, (**II**), and postacquisition processing (**III**, and **IV**). Using the single scan FIDs (**II**) and implementing the signal averaging in postacquisition processing (**III)**, increases the number of kinetic data points, *N*_(*n*)_, compared to conventional averaging (**I**), with the same beneficial effect on the S/N in the spectra that they are derived from. Conventional reaction monitoring (**I**) generates the first and the last kinetic data points from two sets of spectrometer-averaged scans, one at the beginning and one at the end of the reaction. Using postacquisition processing these two termini can be evaluated in more detail through use of averaging windows that progressively contract in span from *n*–1 to 1 (**IV**). While the S/N will be inherently lower in these two outer regions, the concentrations are still quantitatively described. Overall, the postacquisition approach then yields a total of *N*_(*n*)_^con^ NMR spectra and thus kinetic data points. As *n* is raised, the increase in temporal resolution relative to conventional in-acquisition becomes very significant.^19^

The examples presented in Sections 2.6, 2.7, and 2.8 below use this additional contracting averaging to generate the kinetic data points from *N*_(*n*)_^con^ individual NMR spectra. The final example, presented in Section 2.9, does not. The first and third examples show how the method increases the number of kinetic data points and S/N, and allows flexible optimization of *n* to obtain best data fidelity. The second demonstrates the use of a “total reaction spectrum” to identify the signal from a low concentration intermediate, and then application of the temporal-averaging to analyze the kinetics of the process. The final example shows how the postacquisition FID processing can be applied in a manner that is coherent with phase cycling. This is of particular benefit for faster reactions, where the use of conventional averaging in multiples of e.g., 8 scans can significantly reduce the number of kinetic data points.

### 2.6 Temporal Resolution and Overaveraging

**Figure 4:**
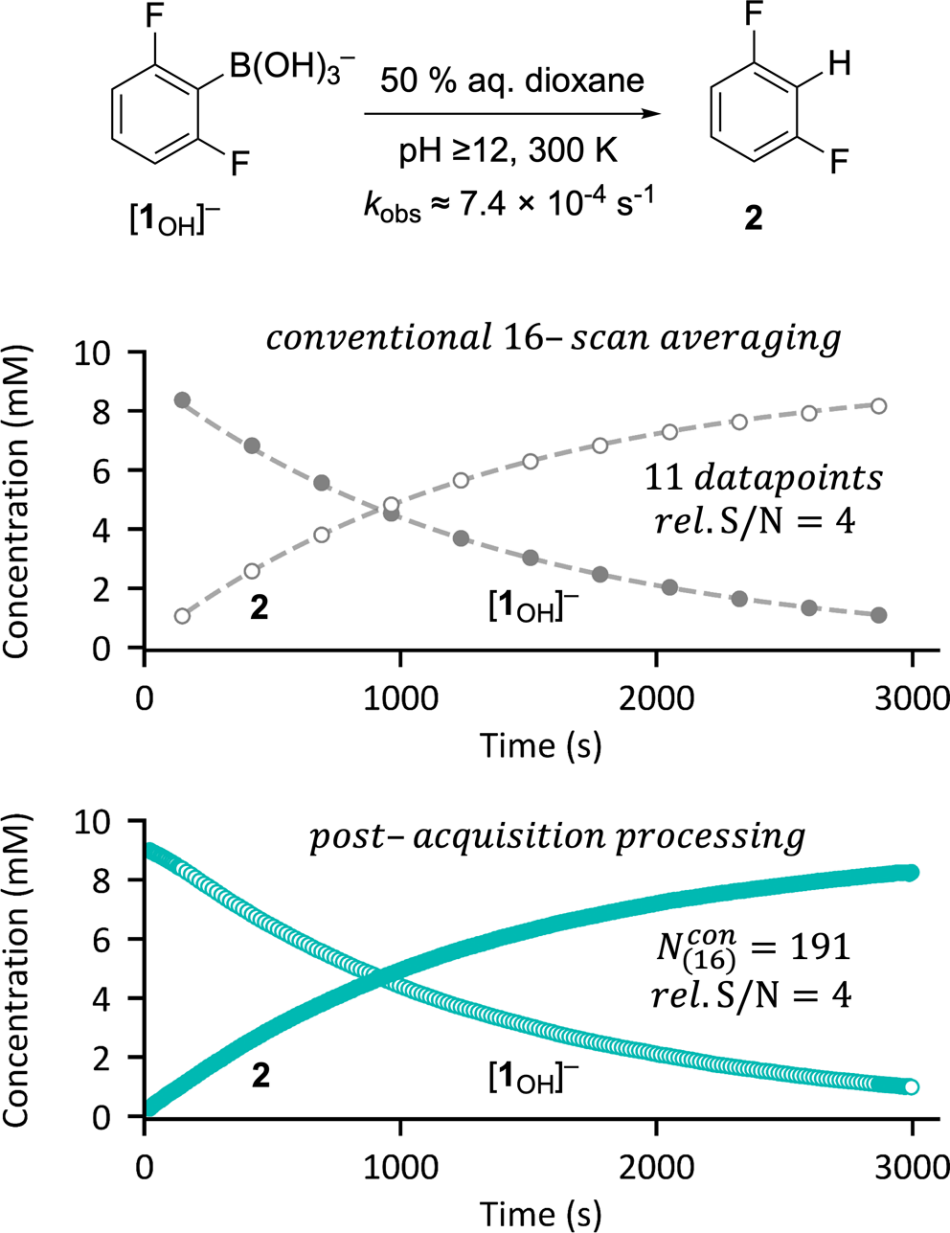
Comparison of ^19^F NMR reaction monitoring data for the protodeboronation of [**1**_OH_]^−^ at 27 °C using conventional on-spectrometer 16-scan averaging (upper) versus postacquisition processing (pathway A, θ = 90°; τ_R_ = 17 s) with contracting averaging (*N*_(16)_^con^ = 191, lower) used to extend the data point range.

The protodeboronation^20^ of 2,6-difluorophenylboronate [**1**_OH_]^−^ to generate 1,3-difluorobenzene **2** proceeds cleanly to completion^21^ over a period of about 1.5 h at 27 °C and high pH. The first-order kinetics are conveniently monitored by *in situ*^19^F NMR spectroscopy, and the slowest relaxing ^19^F nuclei are those in the product, **2**, *T*_1_^max^ = 3.4 s.^11c^ Following Figure 2 leads to selection of pathway A, θ = 90° and τ_R_ = 17 s. A total of 176 single scan FIDs were acquired, and then processed using *n* = 16 averaging, augmented by contracting averaging at the termini. This affords the same S/N as conventional in-acquisition averaging, but with a substantial increase in kinetic data density, *N*_(16)_^con^ = 191, Figure 4.

**Figure 5:**
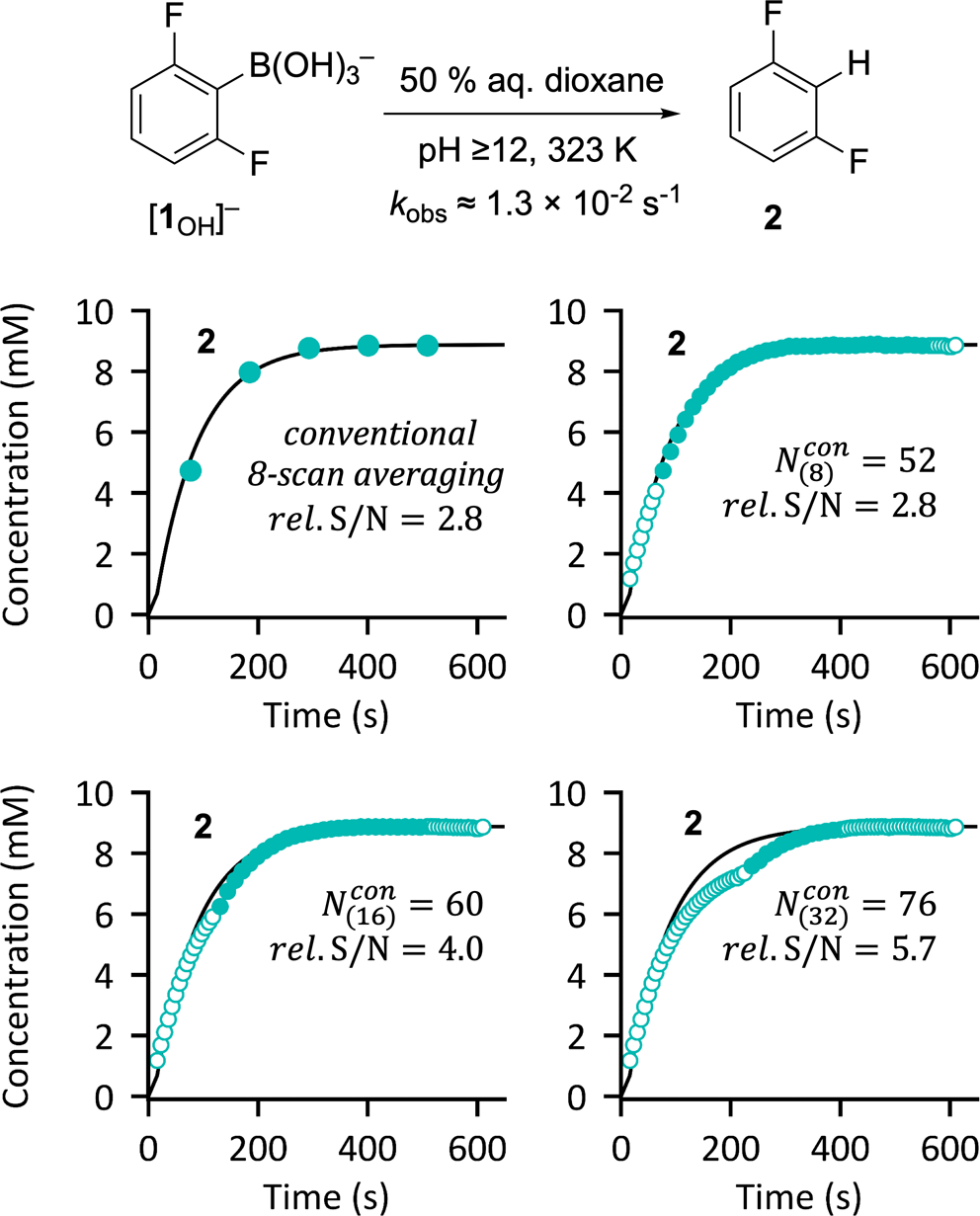
^19^F NMR reaction monitoring data for the protodeboronation of [**1**_OH_]^−^ at 50 °C. Conventional 8-scan averaging versus postacquisition averaging (*N*_(*n*)_^*con*^, Pathway B, θ = 23°; τ_R_ = 13.5 s; *n* = 8, 16, 32). The true kinetic profile for **2** is shown with a solid line; in this case, overaveraging is evident when *n* = 16 and 32.

Conducting the same reaction at 50 °C leads to an increase in *T*_1_^max^ from 3.4 to 5.4 s, and an 18-fold increase in protodeboronation rate. The 6.6 min lifetime^21^ of [**1**_OH_]^−^ leads to selection of Pathway B, θ = 23° and τ_R_ = 13.5 s, and 45 single-scan FIDs were acquired after initiating the reaction,^22^Figure 5. Under these conditions, postacquisition processing using too large a temporal-window (*n* × τ_R_) leads to significant deviations from the true kinetic profile. The onset of this “overaveraging” is evident when *n* = 16, and becomes substantial when *n* = 32, especially at the most curved sections of the kinetic profile. Conversely, for this specific example, applying *n* = 8 provides sufficient kinetic fidelity, and substantial increase in temporal resolution over the conventional on-spectrometer averaging method. Overaveraging is more pronounced when monitoring reaction intermediates, especially in the region of their maximum concentration, where there is an inflection in the temporal concentration gradient; see the spreadsheet in the Supporting Information.

Another form of overaveraging can arise when the weighting of the chemical shifts of two or more species that are in very rapid equilibrium changes during the reaction, for example as a function of evolving pH. Analogous effects can arise through changes in the reaction medium, or lock signal.^2c,4c^ In all cases, the line-shape of these “drifting peaks” will progressively flatten as the temporal-window of averaging is increased, resulting in an apparent loss of coupling, and possible overlap with signals from other species.

Identical overaveraging effects arise in the conventional method (**I**, Figure 3). However, since the scans are precombined on the spectrometer during reaction monitoring, the impact of overaveraging on the fidelity of the temporal concentration data cannot be mitigated after the NMR spectra have been acquired. In contrast, being able to flexibly vary the temporal-window (*n* × τ_R_) during postacquisition processing (**III**, **IV**), allows overaveraging effects to be identified and attenuated without the need for further experiments. Indeed, the signal averaging can even be adjusted to generate additional temporal concentration datasets, optimized for individual species.

### 2.7 Locating Intermediates in a Total Reaction Spectrum, *N*_(∑)_

**Figure 6:**
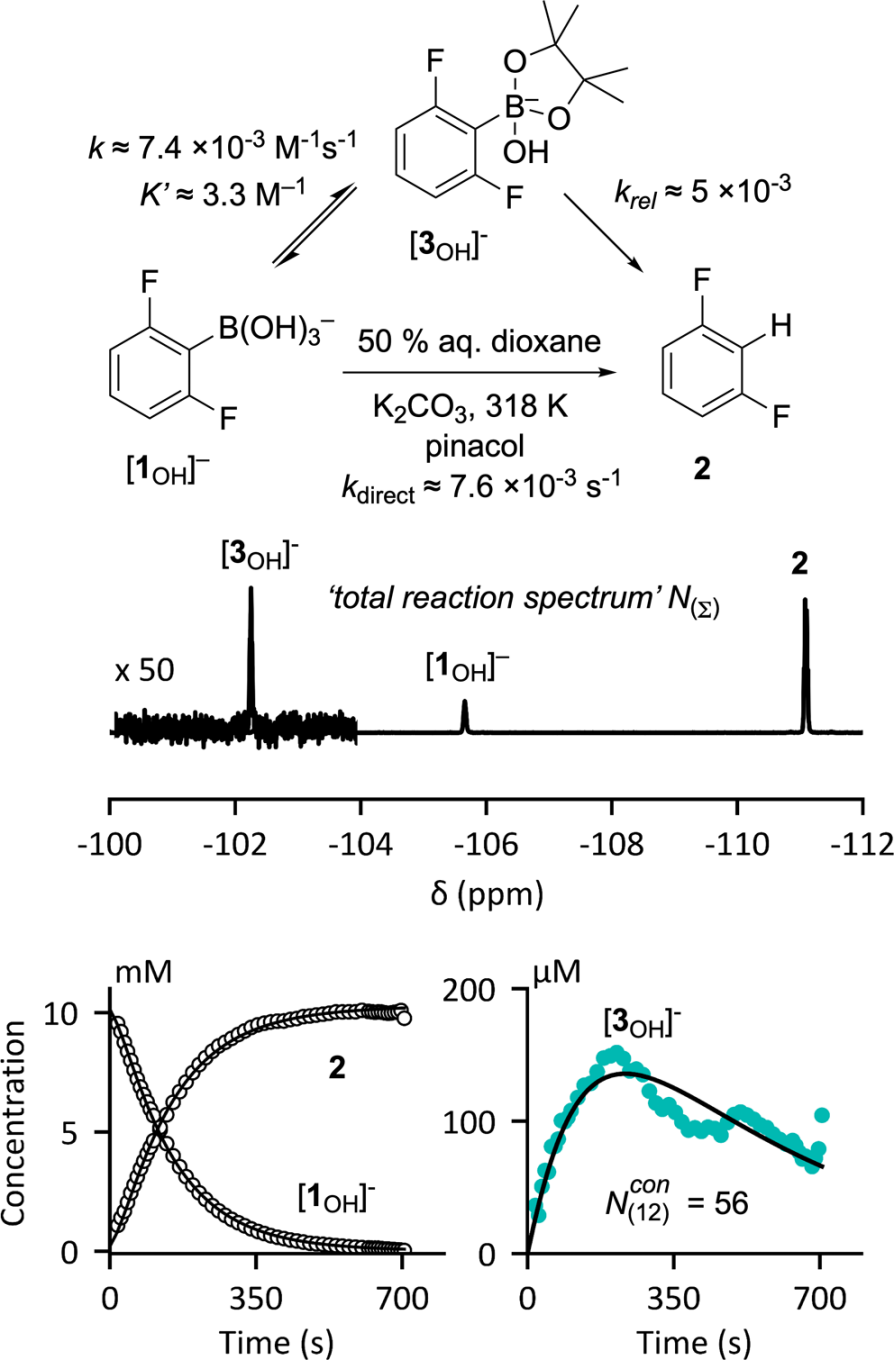
^19^F NMR reaction monitoring of the protodeboronation of [**1**_OH_]^−^ in the presence of pinacol. The “total reaction spectrum” (*N*_(∑)_), produced by averaging all 45 scans, identifies generation of boronate [**3**_OH_]^−^. Using the integral region identified for [**3**_OH_]^−^ and application of postacquisition processing (*N*_**(**12**)**_^con^ = 56) allows kinetic simulation of the system (solid lines through the data); see the Supporting Information Section S3.5.

Conducting the protodeboronation of [**1**_OH_]^−^ in the presence of pinacol allows competitive equilibration with the corresponding boronate ester, [**3**_OH_]^−^,^23^Figure 6.

The reaction was monitored at 45 °C, following Pathway B with θ = 41° and τ_R_ = 16 s over the 12 min reaction lifetime.^21^ The “total reaction spectrum” (*N*_(∑)_) obtained by averaging the full set of 45 scans provides maximum S/N and enables detection of the transient boronate [**3**_OH_]^−^ which reaches a maximum concentration of about 150 μM after about 3 min. Having identified the chemical shift and integral region for [**3**_OH_]^−^, postacquisition processing (*N*_(12)_^con^ = 56) affords data for kinetic modeling, Figure 6, without having to repeat the experiment.

Care must be taken when generating a total reaction spectrum from systems in which spectral crowding or drifting peaks are apparent. Nonetheless, in such cases spectra can still be generated from FIDs created using much larger averaging windows than will be employed for the kinetic data, and the enhanced S/N used to aid identification of integral regions.

### 2.8 Signal/Noise and Data Fitting

Low S/N reduces the reliability of constants and coefficients derived by curve fitting of kinetic data.^1^ This applies for example in the experimental determination of heavy atom KIEs by *in situ* NMR spectroscopy for comparison with those predicted by theory.^4b^ The competitive hydrolysis of isotopically labeled MIDA boronates^24^ [^13^C_2_]-**4**/[^2^H_4_]-**4** (23 mM, *T*_1_^max^= 3.0 s) was monitored by *in situ*^19^F NMR spectroscopy at 50 °C, following Pathway A with θ = 90° and τ_R_ = 24.5 s (8 *T*_1_^max^)^4b^ over the 60 min reaction lifetime.^21^

**Figure 7:**
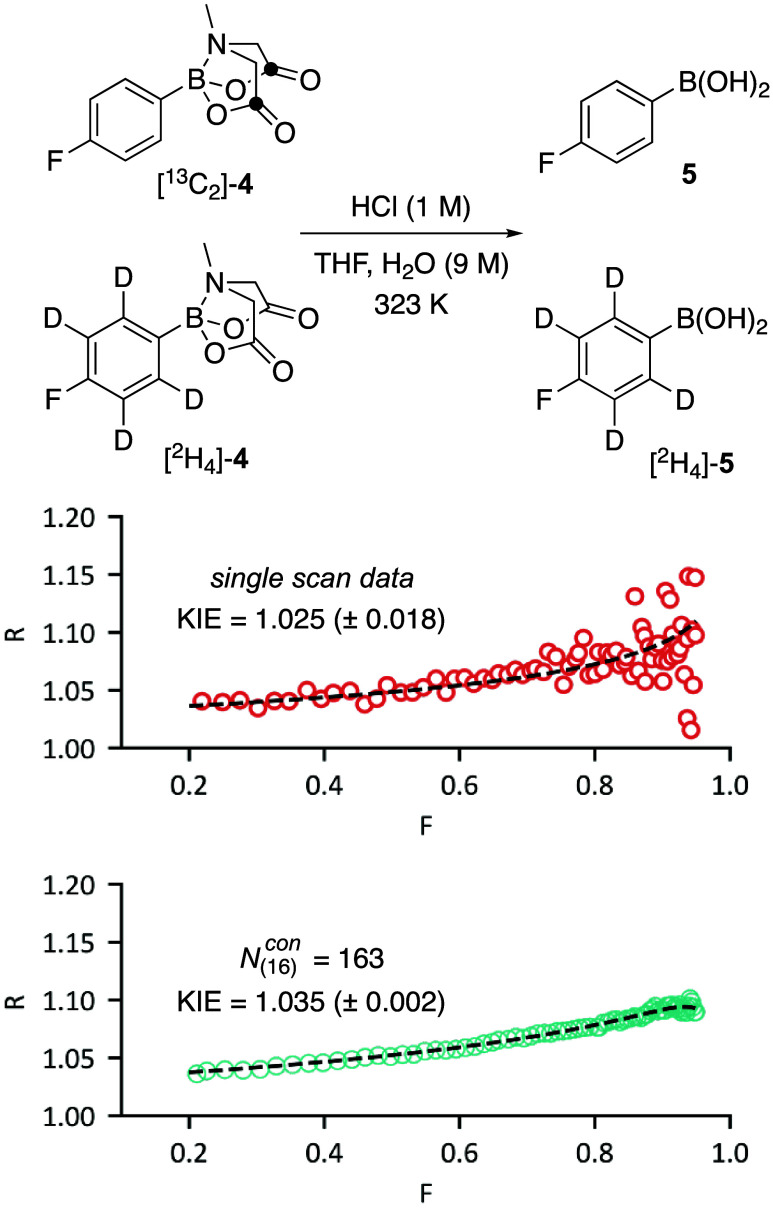
Isotope ratio (*R*) versus fractional conversion (*F*) for the hydrolysis of [^13^C_2_]-**4**/[^2^H_4_]-**4** (*R*_0_ = 1.031) monitored by *in situ*^19^F NMR spectroscopy. The KIEs (±2σ) are from Bigeleisen-Wolfsberg analysis of *R*/*R*_0_ between 0.2 ≤ *F* ≤ 0.95.^4b^ Postacquisition processing allows identification of the impact of an inert contaminant (0.1%) and correction for this in the fitting; see the Supporting Information Section S4. The dashed lines correspond to the (*F*, *R*) values predicted by the fitted KIEs.

The data are analyzed conventionally by the change in substrate isotope ratio, *R*, as a function of fractional conversion, *F*. Postacquisition FID processing (*N*_(16)_^con^ = 163) increases the S/N and yields the KIE with improved accuracy and precision, Figure 7.

### 2.9 Coherence with Phase Cycling and Solvent Suppression

In the examples above, a sequence of single-scan FIDs were collected via a standard pseudo 2D-mode kinetics experiment.^17^ However, in some cases the application of more complex pulse sequences is necessary, for example, when reactions are monitored by ^1^H NMR spectroscopy in nondeuterated solvent. In this case, the application of a solvent suppression pulse sequence^25^ allows the intensity of the dominant signals from the solvent to be substantially attenuated. This then increases the dynamic range available for analysis of the relative intensities of the signals from the analytes of interest, i.e., the reaction components.^1^ Phase-cycling can be implemented to minimize spectral distortions introduced by imperfections in the solvent suppression pulse sequence.^25^

In the conventional reaction monitoring method, the in-acquisition signal averaging is set to be coherent with the phase cycles. Using NMR spectra generated from FIDs acquired without coherence to the full phase cycle results in distortions that are amplified through signal averaging and can render the spectra nonquantitative. Thus, for example when the phase cycling uses 8 scans, a limit of (8 × τ_R_), or multiples thereof, is imposed on the temporal resolution of the kinetic data points. In contrast, by postacquisition processing of single-scan data, and setting *n* set to be an integer-multiple of the number of phases cycled, the number of kinetic data points is increased, without causing spectral distortion.^26^

**Figure 8:**
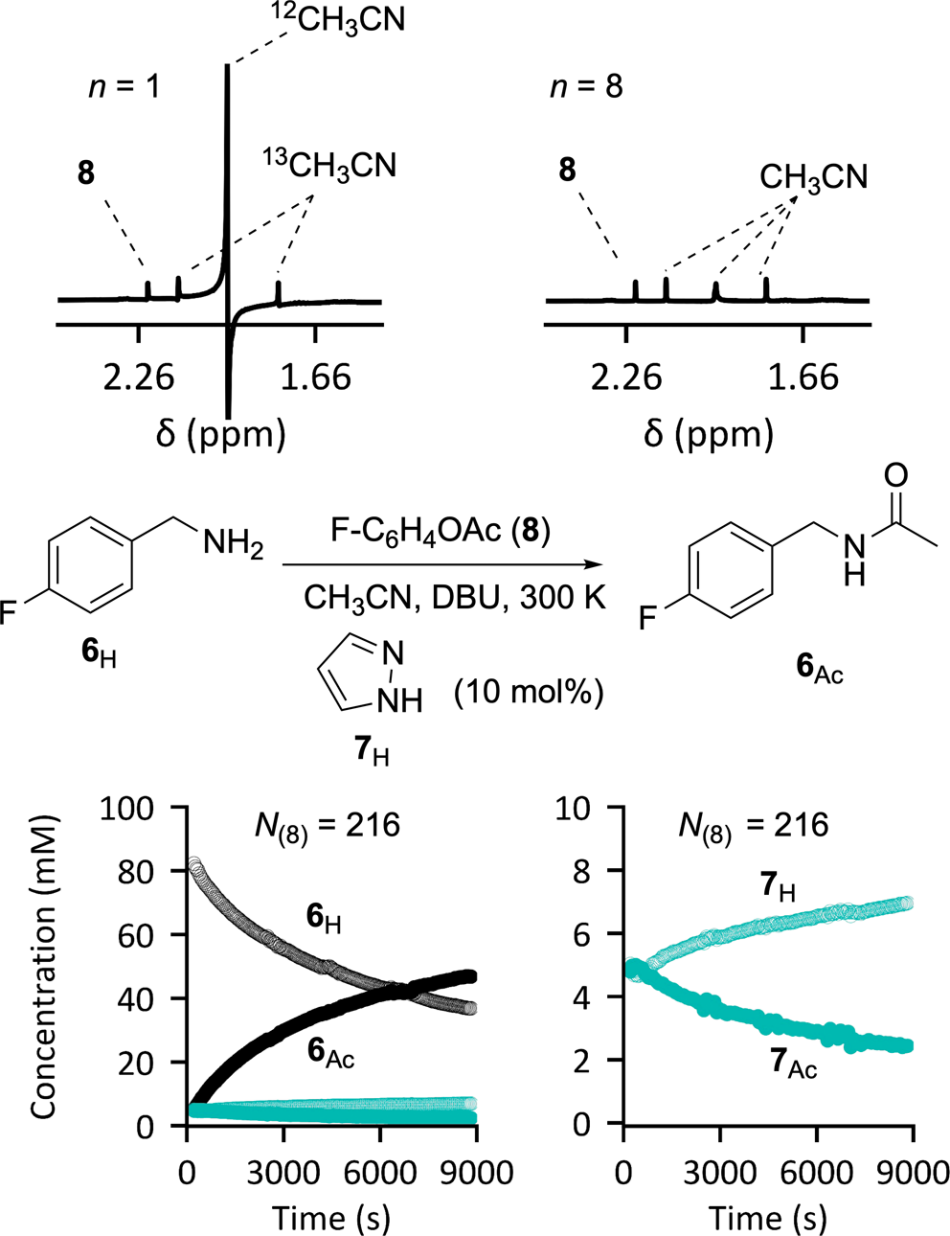
Comparison of postacquisition processed ^1^H NMR subspectra for *in situ* reaction monitoring of an azole-catalyzed acylation with (*n* = 8) and without (*n* = 1) coherence to the phase cycling. Correct selection of the averaging window (*N*_(8)_ = 216) allows ^1^H NMR analysis of the reaction (**6**_H_ → **6**_Ac_) and the temporal speciation of the catalyst intermediates (**7**_H_ and **7**_Ac_) in nondeuterated solvent, CH_3_CN.

To illustrate this concept, the azole-catalyzed^27^ acylation of amine **6**_H_ was carried out in nondeuterated acetonitrile, CH_3_CN, and monitored by ^1^H NMR spectroscopy using solvent suppression with a standard phase-cycling routine.^25^ Without applying coherent signal averaging, significant residual solvent signals are present, and the resulting distortions render the spectra intractable to global phasing,^28^ e.g., *n* = 1 in Figure 8.

When postacquisition averaging is coherent with the overall phase cycle (in this specific case, *n* = 8), the solvent suppression is highly effective, and the distortions eliminated. While the phase cycling constrains the averaging window, the number of kinetic data points are still increased compared to conventional in-acquisition averaging, in this case by 8-fold. It is important to note that additional contracting averaging (**IV**) has not been applied to the data in Figure 8 because this leads to incoherence with the phase cycle and spectral distortions.

## 3 Conclusions

Conventional methods for on-spectrometer signal averaging constrain the mutual optimization of S/N and temporal resolution for *in situ* reaction monitoring by 1D NMR spectroscopy. However, this dichotomy arose from limitations to the hardware and computational power available during the development of pulse NMR. In contrast, modern instruments and software readily allow FID signal averaging to be implemented as a postexperiment processing technique.^18^ Thus, acquisition of a large set of single-scan FIDs and application of a moving average over a window of *n*-scans provides equivalent S/N gain from conventional in-acquisition averaging, while maintaining the inherent temporal resolution of the single-scan intervals, τ_R_. Compared to conventional on-spectrometer signal averaging, this enables a greater number of kinetic data points, and more insight into the early and late phases of a kinetic profile, Figure 5.

In parallel, the combination of all of the single scan data into single, “total reaction spectrum”,^29^*N*_(Σ)_Figure 6, provides maximized S/N to aid the identification of species present in relatively low concentrations during the reaction, e.g., intermediates. By suitable choice of averaging window, the postacquisition processing technique can also be applied to more complex pulse sequences involving phase cycling, Figure 8. Postacquisition processing not only maximizes the information available from a single experiment, but also minimizes the risk of overaveraging, Figure 5, and enhances the quality of data for curve fitting, Figure 7. It also simplifies the pre-experiment parameter selection to estimation of the reaction monitoring time and *T*_1_^max^, and then identifying the optimum pulse angle, θ, and scan repetition time, τ_R_, see Figure 2 and the step-by-step guide below.

## 4 Step-by-step Guide

The general work-flow and other key points for consideration are summarized below. See the Supporting Information for full discussion, a link to the video to accompany the step-by-step guide, a spreadsheet for calculation of θ, τ_R_, and time points, and scripts for automation of acquisition and processing.

(i) Estimate the total reaction time, or the time-period to be monitored if the process is being partially analyzed.
(ii) Select a suitable^1a^ internal standard to provide a reference for integration.
(iii) Determine the slowest longitudinal relaxation rate, *T*_1_^max^, of all nuclei to be monitored, including the integration standard. A dummy sample of a noninitiated or partially complete reaction can be useful for these measurements.^1a,11c^
(iv) Estimate the number of kinetic data points required to analyze the process, ensuring to consider whether this will include investigation of intermediates.
(v) Using the spreadsheet identify the pathway (A, B, C, Figure 2), pulse angle, θ, scan repetition time, τ_R_, and whether a single or multiscan FID acquisition mode will be employed. Use the generic temporal concentration plot generated in the spreadsheet to review step iv, and if necessary, repeat step v.
(vi) Parameterize an automated acquisition sequence on the spectrometer, by using one of the provided scripts or asking your NMR facility to generate the pulse program for you.
(vii) Initiate the reaction, acquire the series of FIDs,^17^ and note the deadtime.^1a,22^
(viii) Use the automation script provided or ask your NMR facility to generate one for you, to combine scans to generate new FIDs for Fourier transform (*N*_(*n*)_^con^, II to IV, Figure 3). If there is a phase cycling component to the pulse sequence, do not apply contracted averaging (*N*_(*n*)_, II to III, Figure 3) and ensure that “*n*” is coherent with the number of phase cycles.
(ix) If desired, use the automation script provided to combine all of the original scans in one FID, then Fourier transform and phase to obtain the “total reaction spectrum”,^28^*N*_(Σ)_ and identify regions for integration.
(x) Fourier transform the new FIDs generated in step viii, phase and integrate the resulting time-domain spectra as normal.
(xi) Using the spreadsheet provided, determine the time points to use for each spectrum and thus generate the temporal concentration dataset for analysis.
(xii) Check the temporal concentration dataset for overaveraging and the individual NMR spectra for aberrations such as overlap of drifting peaks, and if required repeat steps viii, x and xi, for selected species.

## Data Availability

The data underlying this study are available in the published article and its Supporting Information. The primary NMR data, pulse programs and scripts are also available free of charge at https://doi.org/10.7488/ds/7827.

## Supporting Information Available

A guide to estimate *T*_1_^max^ (XLSX)
A guide to parameter selection (XLSX)
Pulse sequences and automation scripts (ZIP)
Details on reaction assembly, kinetic analyses, discussion of the impact of basic NMR acquisition parameters on data density and S/N (PDF)
Step-by-step video guide to parameters and acquisition (MP4)
Step-by-step video guide to processing and installing scripts (MP4)
