# Supplementary material for: Simple Parameters and Data Processing for Better Signal-to-Noise and Temporal Resolution in In Situ 1D NMR Reaction Monitoring
Source: J Org Chem. 2024 Nov 1;89(22):16586–93. doi: 10.1021/acs.joc.4c01882 (PMC11574850; doi:10.1021/acs.joc.4c01882)
Supplement: Supplementary file 4 — jo4c01882_si_004.pdf [file jo4c01882_si_004.pdf]

Simple Parameters and Data Processing for Better Signal-to-Noise and Temporal Resolution in *In Situ* 1D NMR Reaction Monitoring

Annabel Flook and Guy C. Lloyd-Jones*

*E-mail: guy.lloyd-jones@ed.ac.uk

For an accompanying video see: https://youtu.be/oasIsNiWfts

SUPPORTING INFORMATION
Table of Contents

S1. General Experimental Methods – NMR, Suppliers and Exp S3

S1.2. NMR Experiments S3

S1.3. General Reaction Monitoring – Protodeboronation Reactions....................S3

S2. Temporal Resolution and Over-averaging S4

S2.1. Stock Solutions S4

S2.2. ^{19}F T_1 Measurements – at 300 K and at 323 K S4

S2.3. Reaction Monitoring – At 300 K....................S4

S2.4. Processing Parameters – At 300 K S5

S2.5. Reaction Monitoring – At 323 K....................S5

S2.6. Processing Parameters – At 323 K S5

S3. Locating Intermediates in a Total Reaction Spectrum, $N_{(\Sigma)}$ S7

S3.1. Stock Solutions S7

S3.2. ^{19}F T_1 Measurements – At 318 K S7

S3.3. Reaction Monitoring – At 318 K....................S7

S3.4. Processing Parameters....................S8

S3.5. Protodeboronation: Kinetic Simulation S8

S4. Signal/Noise and Data Fitting S9

S4.1. Stock Solutions S9

S4.2. ^{19}F T_1 Measurements – At 323 K S9

S4.3. Reaction Monitoring – At 323 K....................S9

S4.4. Processing Parameters....................S10

S5. Averaging for Phase Cycling and Solvent Suppression. S11

S5.1. Stock Solutions S11

S5.2. Reaction Monitoring S11

S5.3. Processing S11
S6. Pulse Programs..........S12
S6.1 Pulse Program Template for Phase Cycling - With Annotations S12
S6.2. Pseudo-2D Pulse Program with Pre-Saturation Solvent Suppression and Phase Cycling – With Annotations..........S12
S7. Discussion on adjusting time points to reflect averaging..........S14
S8. Pathway B: Reduction of τ_R and θ Increases Signal-to-Noise in Some Scenarios..........S16
S9. Pulse Angles, the Ernst Equation and Quantitative Parameters S19
S10. Data Processing Validation S20
S11. - TopSpin 4.3.0 AU Programs for Averaging – With Annotations..........S21
S11.1. Averaging of FIDs S21
S11.2. Averaging of FIDs with Additional Contracting..........S22
References S25

Supplementary Information

S1. General Experimental Methods – NMR, Suppliers and Exp

Unless otherwise stated, reagents were purchased from commercial sources (Sigma Aldrich or Fluorochem) at the highest available grade, and were used without purification. All internal standards used for kinetic monitoring experiments were of ≥ 99% purity.

Dioxane was introduced and stored in the glovebox from the time of purchase to minimise peroxide formation. All stock solutions were prepared in volumetric glassware, under ambient conditions, outside of the glovebox and typically used on the day of their preparation. All solutions stored for longer periods (< 3 days) had their stability/composition assessed by ^{1}H NMR analysis directly prior to use in reaction monitoring experiments preparation.

Anhydrous MeCN was obtained by passing it through a column of anhydrous alumina using an Anhydrous Engineering Grubbs-type system and storing over 3 Å molecular sieves for > 48 h. Molecular sieves were activated by heating *in vacuo* (220 °C, 0.8 mbar) for > 16 h.

For stock solutions using dioxane or MeCN as solvent, solids were weighed into volumetric glassware, which was septum sealed and purged with N_2. Solvent was transferred *via* syringe into the volumetric flask. During reaction monitoring, no special precautions were made to exclude O_2. *At the low concentrations employed, some of the boronic acid is oxidized by peroxides, or air, in the dioxane, to generate the corresponding phenolate, on addition of the base.*

S1.2. NMR Experiments

NMR spectra obtained for characterisation were acquired on a Bruker Ascend 400 MHz NMR spectrometer fitted with a broadband direct-detect Cryoprobe Prodigy.

S1.3. General Reaction Monitoring – Protodeboronation Reactions

S1.3.1. ^{19}F T_1 Measurements

T_1 measurements for trifluoroacetic acid and [**2**] were recorded using FLIPS[S1] on a sample, prepared identically to the monitored sample, and allowed to react to completion. Measurements were recorded on the same day as, and prior to, the reaction monitoring experiments and at the same temperature. Boronate $[\mathbf{1}_{OH}]^-$ is unstable to protodeboronation under the reaction conditions. The T_1 of boronic acid [**1**] in dioxane was measured as a surrogate for the unstable species. All T_1 measurement data were processed by using Bruker TopSpin 3.6.5 and MestReNova (Version 15.0.0).

S1.3.2. Reaction Monitoring

Prior to reaction monitoring, a reaction was initiated in an NMR tube in the same manner as the intended reaction monitoring sample, and was allowed to react to completion. The probe was set to maintain the desired temperature during reaction monitoring. Using this pre-reacted sample, the NMR probe was tuned to ^{1}H and matched and subjected to gradient shimming (lockoff ^{1}H). The probe was then tuned and matched to ^{19}F.

Using the approximated monitoring time and the T_1^{max} for the reacting species, the flowchart (Figure 2, main text) and spreadsheet (Supplementary Information) was used to determine NMR parameters for reaction monitoring. To initiate the reaction, stock solutions were combined in a new NMR tube, vigorously shaken and placed back into the probe.

S1.3.3. Data Processing

All FID and spectral processing was performed in TopSpin 4.3.0. Spectra generated through signal-averaging were processed identically to the spectra collected during acquisition to allow for comparison. Unless otherwise stated, spectra were Fourier transformed without window functions. Zero and first order phase correction values were determined manually and remained consistent throughout. An automatic baseline correction was applied between regions -100 ppm to -115 ppm and between -70 ppm and -80 ppm. Integral regions were selected manually. Phase correction values and integral regions are stated for each experiment.

S2. Temporal Resolution and Over-averaging

S2.1. Stock Solutions

Concentrations of reacting species were intentionally selected to provide systems in which signal intensity is low. The following stock solutions were made.

Stock Solution A1: trifluoroacetic acid (0.42 mL, 626 mg, 5 mmol) was made to 10 mL with H_2O.
Stock Solution A2: KOH (115 mg, 2 mmol) and Stock Solution A1 (2 mL) was made to 5 mL with H_2O.
Stock Solution A3: Stock Solution A2 (1 mL) was made to 5 mL with H_2O.

Stock Solution B1: 2,6-difluorophenyl boronic acid (69 mg, 0.6 mmol) was made to 5 mL with 1,4-dioxane.
Stock Solution B2: Stock Solution B1 (1 mL) was made to 5 mL with 1,4-dioxane.

Reaction: Stock Solution A3 (0.3 mL) and Stock Solution B2 (0.3 mL) combined in an NMR tube.
Reactant Concentrations: KOH (20 mM), 2,6-difluorophenyl boronate (12 mM), TFA (20 mM).

S2.2. ^{19}F T_1 Measurements – at 300 K and at 323 K

Table S1. *^{19}F T_1 Measurements for trifluoroacetic acid and [**2**] under reaction conditions and [**1**] in dioxane.*

		***T_1 (s),* at 300 K**	***T_1 (s),* at 323 K**
Trifluoroacetic acid (Internal Standard)	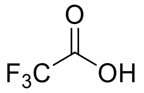	**1.8**	**2.1**
[**2**]	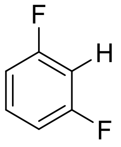	**3.4**	**5.4**
[**1**]	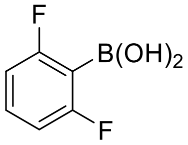	**2.1**	**2.9**

S2.3. Reaction Monitoring – At 300 K

Using the Flowchart (Figure 2, main text) and spreadsheet (Supplementary Information). The T_1^{max} was set at 4.6 s and the reaction monitoring time set to 3000 s. Pathway A was used to calculate parameters for NMR monitoring: θ = 90°, τ_R = 17 s, resulting in 176 datapoints. Monitoring was commenced 20 s after mixing of stock solutions A3 and B2 to initiate the reaction, and 176 single scan spectra were collected over 3000 s. The parameters used for monitoring are given in Table S2.

Table S2. *NMR Reaction Monitoring Parameters, as calculated from spreadsheet, and as used experimentally.*

**Parameters**	**Calculated (from spreadsheet)**	**Experimental Parameters**
Chosen Pulse Angle (θ)	90	90
Number of scans per spectrum	1	1
Repetition Time ($\tau_{R,}$ s)	16.95	17 (including acquisition time)

S2.4. Processing Parameters – At 300 K

In post-acquisition processing, a contracting average was applied ($N_{(16)}^{con} = 191$). Zero and first order phase correction were set to -142.400 and 7.200 respectively. Integration of all spectra were carried out between the following ranges:

Table S3. *Integration parameters used for raw data and averaged data.*

^{19}F Resonance	Integral Minimum (ppm)	Integral Maximum (ppm)
TFA (Internal Standard)	-75.692	-74.756
[$\mathbf{1}_{OH}$]$^-$	-105.840	-105.064
[**2**]	-111.048	-110.361

The time axis was calculated using the 'Averaging Time' tab in the spreadsheet, with Deadtime = 20 s, Repetition Time = 17 s, Total Number of Acquired Spectra = 191, Averaging Block Size = 16 and Averaging Type = Contracted. The conventional 16-scan averaging data was generated using every 16th spectrum from the contracting averaging dataset.

A simple first-order kinetic model was fit to the single scan data, with the following parameters: [$\mathbf{1}_{OH}$]$^-_0$ = 9.05 mM, [**2**]$_0$ = 0.274 mM, k_{obs} = 7.38 × 10^{-4} s^{-1}.

S2.5. Reaction Monitoring – At 323 K

Using the Flowchart (Figure 2, main text) and spreadsheet (Supplementary Information), the collection of 45 spectra was deemed to provide appropriate data density. The T_1^{max} was set at 5.4 s and reaction lifetime was estimated at 600 s. Monitoring was commenced 16 s after mixing.

Table S4. *NMR Reaction Monitoring Parameters, as calculated from spreadsheet, and as used experimentally.*

Parameters	Calculated (from spreadsheet)	Experimental Parameters
Chosen Pulse Angle (θ)	23	23
Number of scans per spectrum	1	1
Repetition Time ($\tau_{R,}$ s)	13.3	13.5 (including acquisition time)
Number of data points with the smaller pulse angle (N)	45	45

S2.6. Processing Parameters – At 323 K

A contracting average of size 8, 16 and 32 was applied to the raw data to generate a total of 4 series of experiments. The total number of spectra ($N_{(n)}^{con}$) after applying a rolling average size of n to a number of single scan FIDs acquired follows Equation S1.

$$N_{(n)}^{con} = Number\ of\ Single\ Scan\ FID + (n - 1) \qquad \text{Equation S1}$$

Table S5. *Number of resulting spectra after contracting averaging.*

Experiment	Number of Spectra
Raw Spectra	45
$N_{(8)}^{con}$	52
$N_{(16)}^{con}$	60
$N_{(32)}^{con}$	76

Zero and first order phase correction were set to 224.938 and 3.239 respectively. Integration of all spectra were carried out between the following ranges:

Table S6. *Integration parameters used for raw data and averaged data.*

^{19}F Resonance	Integral Minimum (ppm)	Integral Maximum (ppm)
TFA (Internal Standard)	-75.692	-74.756
$[\mathbf{1}_{OH}]^-$	-105.840	-105.064
[**2**]	-111.048	-110.361

The time axis was calculated using the 'Averaging Time' tab in the spreadsheet, with Deadtime = 16 s, Repetition Time = 13.5 s, Total Number of Acquired Spectra = See Table S5, Averaging Block Size = See Table S5 and Averaging Type = Contracted.

S3. Locating Intermediates in a Total Reaction Spectrum, $N_{(\Sigma)}$

S3.1. Stock Solutions

Concentrations of reacting species were intentionally selected to provide systems in which signal intensity is low. The following stock solutions were made.

Stock Solution A1: trifluoroacetic acid (0.42 mL, 626 mg, 5 mmol) was made to 10 mL with H_2O.
Stock Solution A2: Stock Solution A1 (1 mL) was made to 10 mL with H_2O.
Stock Solution A3: K_2CO_3 (80 mg, 0.6 mmol), pinacol (48 mg, 0.4 mmol) and Stock Solution A2 (1 mL) made to 10 mL with H_2O.

Stock Solution B1: 2,6-difluorophenyl boronic acid (34 mg, 0.3 mmol) was made to 10 mL with 1,4-dioxane.

Reaction: Stock Solution A3 (0.3 mL) and Stock Solution B1 (0.3 mL) combined in a NMR tube.
Reactant Concentrations: K_2CO_3 (29 mM), 2,6-difluorophenyl boronic acid (15 mM), Pinacol (20 mM), Trifluoroacetic acid (0.5 mM).

S3.2. ^{19}F T_1 Measurements – At 318 K

The T_1 of boronic acid [**1**] in dioxane was smaller than [**2**] at both 303 K and 323 K and was not remeasured for 318 K. The pinacol boronate intermediate was not stable in the reaction conditions, and T_1 was not measured for this species.

Table S7 *^{19}F T_1 Measurements for trifluoroacetic acid and [**2**] under reaction conditions.*

		**T_1 at 318 K (s)**
Trifluoroacetic acid (Internal Standard)	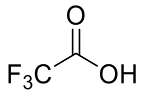	**1.9**
[**2**]	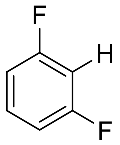	**4.3**

S3.3. Reaction Monitoring – At 318 K

Using the Flowchart (Figure 2, main text) and spreadsheet (Supplementary Information), the collection of 45 spectra was deemed to provide appropriate data density. The T_1^{max} was set at 4.3 s and reaction monitoring time was set as 700 s. Pathway B was used to calculate parameters for NMR monitoring: θ = 41°, τ_R = 16 s. Monitoring was commenced 18.6 s after mixing and 45 spectra were collected over 700 s.

Table S8. *NMR Reaction Monitoring Parameters, as calculated from spreadsheet, and as used experimentally.*

Parameters	Calculated (from spreadsheet)	Experimental Parameters
Chosen Pulse Angle (θ)	41	41
Number of scans per spectrum	1	1
Repetition Time ($\tau_{R,}$ s)	15.5	16 (including acquisition time)
Number of data points with the chosen pulse angle (N)	45	45

S3.4. Processing Parameters

In post-acquisition processing, a contracting average was applied ($N_{(12)}^{con} = 56$) and the total reaction spectrum ($N_{(\Sigma)}$). Zero and first order phase correction were set to 219.654 and 10.229 respectively. The spectra were Fourier transformed with an exponential apodization (line broadening = 1 Hz) window function.

Integration of all spectra were carried out between the following ranges:

Table S9. *Integration parameters used for raw data and averaged data.*

^{19}F Resonance	Integral Minimum (ppm)	Integral Maximum (ppm)
TFA (Internal Standard)	-76.469	-75.575
[$\mathbf{3}_{OH}$]$^-$	-102.966	-102.585
[$\mathbf{1}_{OH}$]$^-$	-106.418	-105.929
[**2**]	-112.199	-111.203

The time axis was calculated using the 'Averaging Time' tab in the spreadsheet, with Deadtime = 18.6 s, Repetition Time = 15.6 s, Total Number of Acquired Spectra = 56, Averaging Block Size = 12 and Averaging Type = Contracted.

S3.5. Protodeboronation: Kinetic Simulation

A simple kinetic model was fitted to experimental data using a standard numerical methods approach[S2] implemented through Excel with a proprietary add-in.

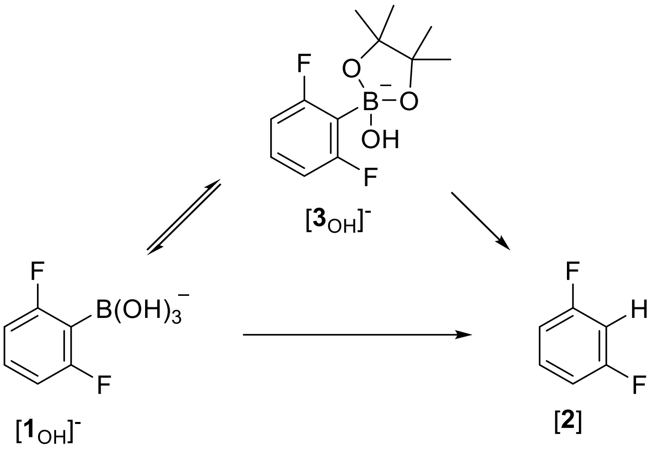

Reaction Process	Rate constant (*k*)	Unit
**[$\mathbf{1}_{OH}$]$^-$ > [2]**	7.64 x 10^{-3}	1/s
**[$\mathbf{1}_{OH}$]$^-$ + pin > [$\mathbf{3}_{OH}$]$^-$**	7.38 x 10^{-3}	L/mol.s
**[$\mathbf{3}_{OH}$]$^-$ > [$\mathbf{1}_{OH}$]$^-$ + pin**	4.51 x 10^{2}	1/s
**[$\mathbf{3}_{OH}$]$^-$ > [2]**	3.40 x 10^{-5}	1/s

Kinetic simulation for protodeboronation of **[$\mathbf{1}_{OH}$]$^-$** in the presence of pinacol (2 equivalents) and base (3 equivalents). A heat transfer constant of k = 0.027 s^{-1} was also included to account for the temperature gradient between the prewarmed samples at mixing (approximated at 303 K) and the reaction mixture at spectrometer temperature (set to 313 K).[2]

S4. Signal/Noise and Data Fitting

S4.1. Stock Solutions

The following stock solutions were made:

Stock Solution A: HCl (1 mL, 37% w/w) was made to 2mL with H_2O.

Stock Solution B: 4-bromobenzotrifluoride (22 mg, 0.1 mmol) made to 10 mL with tetrahydrofuran.

Reaction*:* A mixture of both carbon isotopologues of 4-fluorophenyl MIDA boronate (~ 6mg, ~ 0.024 mmol) were added directly to an NMR tube with Stock Solution B (0.5 mL). Reaction was initiated by addition of Stock Solution A (0.1 mL).
Reactant Concentrations: 4-bromobenzotrifluoride (8 mM), ^{13}C-MIDA boronate (29 mM), ^{12}C-MIDA boronate (28 mM), HCl (0.1 M).

S4.2. ^{19}F T_1 Measurements – At 323 K

Table S10 *^{19}F T_1 Measurements for trifluoroacetic acid and [**2**] under reaction conditions.*

		**T_1 at 318 K (s)**
4-bromobenzotrifluoride (Internal Standard)	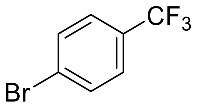	**1.5**
[$^2H_4$]-**4**	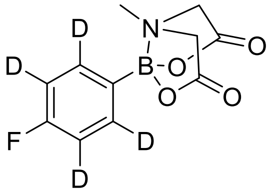	**2.6**
[$^{13}C_2$]-**4**	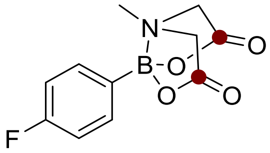	**2.2**
[$^2H_4$]-**5**	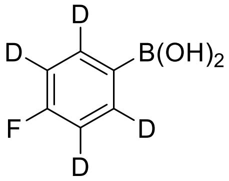	**3.3**
[$^{13}C_2$]-**5**	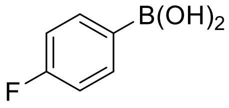	**2.9**

S4.3. Reaction Monitoring – At 323 K

Prior to initiation, NMR tube containing both isotopologues and Stock Solution B (0.5 mL) was heated to 323 K for 10 minutes.

Using the Flowchart (Figure 2, main text) and spreadsheet (Supplementary Information). The T_1^{max} was set at 3.0 s and the reaction monitoring time estimated at 3600 s. Pathway A was used to calculate parameters for NMR monitoring: $\theta = 90°$, $\tau_R = 24.5$ s, with 8 T_1^{max} used for this KIE experiment, resulting in 148 datapoints. Monitoring was commenced 35 s after addition of Stock Solution A (0.1 mL) and vigorous shaking to initiate the reaction. The parameters used for monitoring are given in Table S11.

Table S11. *NMR Reaction Monitoring Parameters, as calculated from spreadsheet Pathway A, and as used experimentally.*

Parameters	Calculated (from spreadsheet)	Experimental Parameters
Chosen Pulse Angle (θ)	90	90
Number of scans per spectrum	1	1
Repetition Time (τ_R, s)	24.00	24.50 (including acquisition time)

S4.4. Processing Parameters

In post-acquisition processing, a contracting average was applied ($N_{(16)}^{con} = 163$). An automatic phasing routine and baseline correction was applied. The spectra were Fourier transformed with an exponential apodization (line broadening = 2 Hz) window function.

Integration of all spectra were carried out between the following ranges:

Table S12. *Integration parameters used for raw data and averaged data.*

^{19}F Resonance	Integral Minimum (ppm)	Integral Maximum (ppm)
Internal Standard	-62.790	-63.662
[$^{13}C_2$]-**5**	-112.191	-112.390
[$^{2}H_4$]-**5**	-112.765	-112.925
[$^{13}C_2$]-**4**	-113.794	-114.062
[$^{2}H_4$]-**4**	-114.357	-114.603

The isotopologue ratio (R) using the concentration data for [$^{13}C_2$]-**4** (B) and [$^{2}H_4$]-**4** (A) were plotted against fractional conversion (F), according to Equations S2 and S3.

$$R = \frac{[B]}{[A]} \quad \text{Equation S2}$$

$$F = 1 - \frac{[A]+[B]}{[A]_0+[B]_0} \quad \text{Equation S3}$$

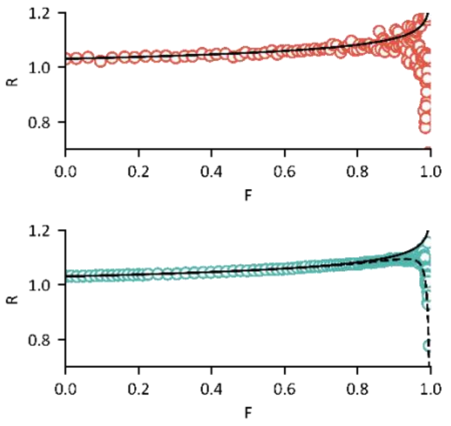

Figure S1*. Isotopologue ratio (R) against fractional conversion (F) from single scan ^{19}F NMR spectra (left). Data were fitted (solid line) to the Bigeleisen-Wolfsberg (BW) equation[S2] in the range F = 0.2 to 0.95 to give the reported KIE (1.025 ±0.018). Following post-processing FID signal averaging ($N_{(16)}^{con}$, right), progressive deviation from the best fit BW equation (KIE 1.028 ± 0.007) is detected as F increases. The data were then re-fitted by accounting for an unidentified inert p-F-C_6D_4X impurity present in the initial reactants, isochronous with [$^{2}H_4$]-**4**. This is consistent with R = 0.78 at F = 0.9956, as measured with higher S/N after the experiment. With the concentration as a variable, a minimum in the standard deviation of the BW calculated list of KIEs in the range F = 0.2 to 0.95 was located (KIE 1.0349 ±0.0018) when the impurity is initially present at 0.11 %. The dashed line (apparent R versus F) was then calculated using a standard numerical methods approach.*

S5. Averaging for Phase Cycling and Solvent Suppression.

S5.1. Stock Solutions

Non-deuterated Stock Solvent: Trimethoxybenzene (132 mg, 0.79 mmol) was made to 25 mL with MeCN.

Solution A: Pyrazole $\mathbf{7}_H$ (2.34 mg, 0.034 mmol), DBU (50 µL, 51 mg, 0.34 mmol) and 4-fluorobenzylamine $\mathbf{6}_H$ (57 µL, 62.4 mg, 0.50 mmol) made to 5 mL with Stock Solvent.

Solution B: 4-fluorophenyl acetate **8** (130 µL, 153 mg, 1.0 mmol) made to 5 mL with Stock Solvent.

Reaction: Solution A (0.6 mL) and Solution B (0.2 mL) combined in a NMR tube.
Reactant Concentrations: Pyrazole (5 mM), DBU (50 mM), 4-fluorobenzylamine (75 mM), 4-fluorophenyl acetate (50 mM), Trimethoxybenzene (Internal Standard, 0.03 mM)

S5.2. Reaction Monitoring

An automatic routine was used to calculate the shape pulse and spectrum centre with a standard NOESY presaturation solvent suppression experiment. The probe was set to maintain 300 K during reaction monitoring. To initiate the reaction, the sample (Solution A) was removed and Solution B (0.2 mL) was added to the NMR tube, vigorously shaken and placed back into the probe. The sample was re-shimmed and monitoring was commenced 94 s after mixing, and a total of 224 spectra were acquired. T_1^{max} was estimated at 8 s based on previous literature,[S3] but was not specifically remeasured in MeCN for the purpose of this case study.

Monitoring was carried out with a pseudo-2D experiment, in which the pulse program is modified to save individual scans to the disk *and* increment the phase pointer associated with the pulse sequence. The 1D solvent suppression pulse sequence and the pseudo-2D solvent suppression experiment are included *vide infra* with annotations to illustrate the differences between them.

Table S13. *NMR Reaction Monitoring Parameters, as calculated from spreadsheet, and as used experimentally.*

Experimental Parameters	Calculated (from spreadsheet)	Experimental Parameters
Chosen Pulse Angle (θ)	90	90
Number of scans per spectrum	1	1
Repetition Time ($\tau_{R,}$ s)	40.0	40 (including acquisition time)

S5.3. Processing

In post-acquisition processing, a moving average coherent with the phase cycling length, 8, was applied ($N_{(8)} = 216$). Note that, due to phase the cycling requirement, all averaged scans must be comprised of 8 single scan spectra (or a multiple thereof). As such, using the contracted average processing will produce poor results for the first and last 7 spectra. The average processing without contraction is thus preferred.

Zero and first order phase correction were set to 197.645 and 1.757 respectively. The spectra were Fourier transformed with an exponential window function of 0.5 Hz, and an automatic baseline correction was applied to the whole spectrum.

S6. Pulse Programs

The pulse programs below have not been widely tested, use at your own risk

S6.1 Pulse Program Template for Phase Cycling - With Annotations

1	**ze**<br>**30m**	
2	**DELTA**	Delay between sequential scans
3	**ipp1**<br>**ipp31**<br>**lo to 3 times c**<br>**ivc**	*ippX* used to increment phase pointer *X* by one. Every phase pointer must be explicitly listed.<br><br>*lo to 3 times c* loops block 3 by *c* times. *c* is defined in a *VCLIST* containing a list of incrementing numbers from 1 to n, where n is the size of the required phase cycle. *ivc* increments the position in *VCLIST*.<br>*See TopSpin Acquisition User Manual (Version 020), VCLIST, for more information on ivc.*
4	**d1**<br>**p1 ph1**<br>**go=3 ph31**<br><br>**30m wr #0 if #0 zd**<br>**lo to 2 times td1**	Pulse sequence<br><br><br><br>Pseudo-2D data saved to disk.

S6.2. Pseudo-2D Pulse Program with Pre-Saturation Solvent Suppression and Phase Cycling – With Annotations

	**;zg2d**<br>**;avance-version (12/01/11)**<br>**;pseudo 2D sequence**<br>**;**<br>**;$CLASS=HighRes**<br>**;$DIM=2D**<br>**;$TYPE=**<br>**;$SUBTYPE=**<br>**;$COMMENT=**<br><br>**prosol relations=<lcnmr>**<br>**#include <Avance.incl>**<br>**#include <Delay.incl>**<br><br><br>**"d12=20u"**<br>**"d13=4u"**<br>**"d20=d19-((td*dw)+d1)"**<br>**"if (d1/p18 < 1) {l6=1;} else {l6=d1/p18;}"**<br>**"l7=trunc(d8/p18)"**<br>**"DELTA=d8-(p18*l7)"**<br>**"acqt0=-p1*2/3.1416"**	Variable definitions
1	**ze**	

	30m	
2	d20	
3	ipp1<br>ipp2<br>ipp3<br>ipp31<br>lo to 3 times c<br>ivc	*Incrementing phase pointers*
4<br><br><br><br><br><br><br><br><br><br>5	p18:sp6:f1 ph29<br>4u<br>lo to 4 times l6<br>d12 pl1:f1<br>p1 ph1<br>d13<br>p1 ph2<br>4u<br>DELTA<br><br>p18:sp6:f1 ph29<br>4u<br>lo to 5 times l7<br>4u pl1:f1<br>p1 ph3<br>go=2 ph31<br><br>30m wr #0 if #0 zd<br>lo to 2 times td1<br>exit	Pulse sequence<br><br><br><br><br><br><br><br><br><br><br><br><br><br><br><br><br>Pseudo-2D data saved to disk.
	 ph1=0 2<br>ph2=0 0 0 0 0 0 0 0 2 2 2 2 2 2 2 2<br>ph3=0 0 2 2 1 1 3 3<br>ph29=0<br>ph31=0 2 2 0 1 3 3 1 2 0 0 2 3 1 1 3<br><br>;d20: D19-(AQ+D1): delay between start of each pulse sequence (ie end of acquisition to start of presat)<br>;td1: number of experiments<br><br>;sp6: f1 channel - shaped pulse for presaturation<br>;p1 : f1 channel - 90 degree high power pulse<br>;p18: f1 channel - presaturation using shaped pulse<br>;d1 : relaxation delay; 1-5 * T1 [ca. 1-2sec]<br>;d8 : mixing time [ca. 80 msec]<br>;d12: delay for power switching [20 usec]<br>;d13: short delay [4 usec]<br>;d19: Delay between acquisitions<br>;l6: p18 * l6 = total duration of presaturation<br>; during relaxation delay [16]<br>;l7: p18 * l7 = total duration of presaturation<br>; during mixing time [1]<br>;ns: 8 * n, total number of scans: NS * TD0<br>;ds: 4<br>;use 100msec pulse of square shape defined by 1000 points<br>;$Id: lc1pnps,v 1.16 2013/02/05 10:11:25 ber Exp $	Phase pointers<br><br><br><br><br><br>Description of variables.

S7. Discussion on adjusting time points to reflect averaging

The average concentration of some species, A, (denoted $\overline{[A]}$) between time points, t_1 and t_2, (assuming continuous data for simplicity of discussion) is defined in Equation S4. To calculate $\overline{[A]}$ for a time point equidistant from t_1 and t_2, a function ($[A]_t$) that describes the change in concentration over time must be known. This is generally not simple, and is made more challenging as the rate equations defining the kinetics of a system are often unknown at the time of collecting and processing reaction monitoring data.

$$\overline{[A]} = \frac{1}{t_2 - t_1} \int_{t_1}^{t_2} [A]_t \, dt \qquad \text{Equation S4}$$

However, if the data between t_1 and t_2 can be approximated as linear, $\overline{[A]}$ is simply the arithmetic mean of data between $[A]_{t_1}$ and $[A]_{t_2}$. This approximation is used for signal-averaging (both on-spectrometer averaging and during post-acquisition averaging). The timepoint $\bar{t}$ associated with $\overline{[A]}$ between t_1 and t_2 is the arithmetic mean of the timepoints between t_1 and t_2.

If this averaged timepoint is not accounted for, but instead assumed to be t_1, the averaged data point will be left-shifted on the x-axis (time). When applying signal-averaging during acquisition, the impact of failing to correct the time axis becomes increasingly apparent as the averaging size is increased. The original data is shown in Figure S2A, and this data with increasing averaging sizes and uncorrected time axis (Figure S2 B-D, left). When corrected, (Figure S2 B-D, right), the datapoints align with the true kinetics (black line). Figures S2 E and F show the same data with contracting and moving averages applied and time corrected. This correction is calculated in the spreadsheet attached, under the tab 'Averaging Time'.

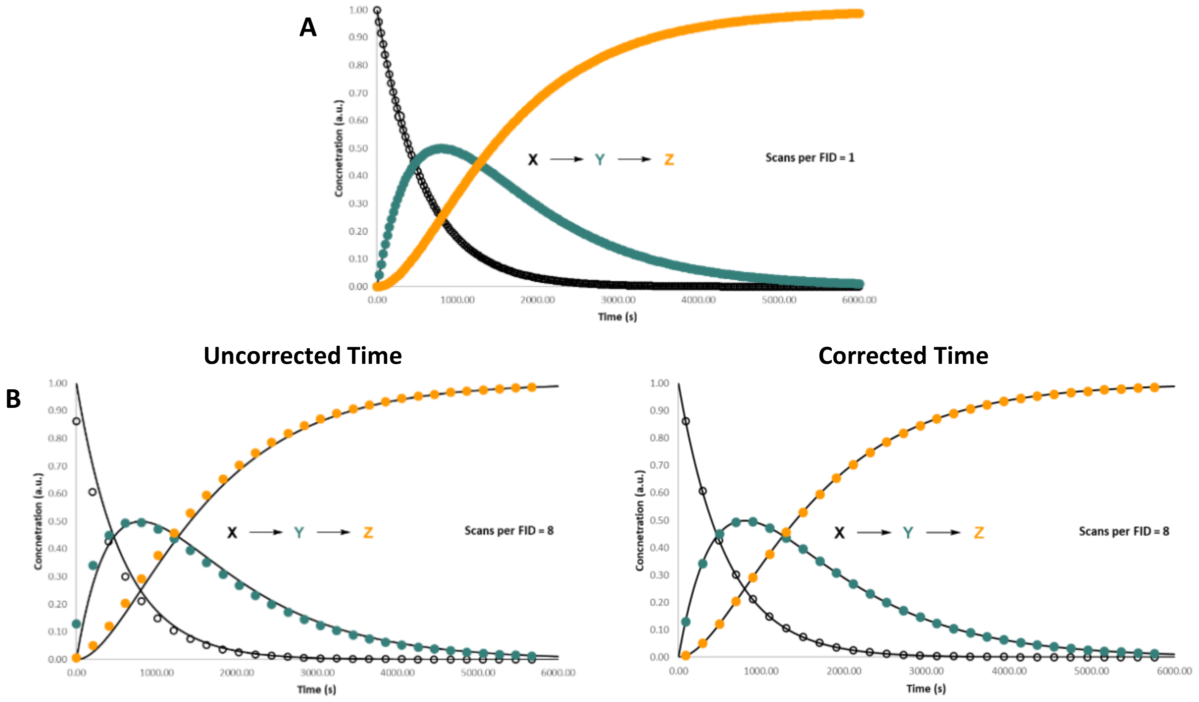

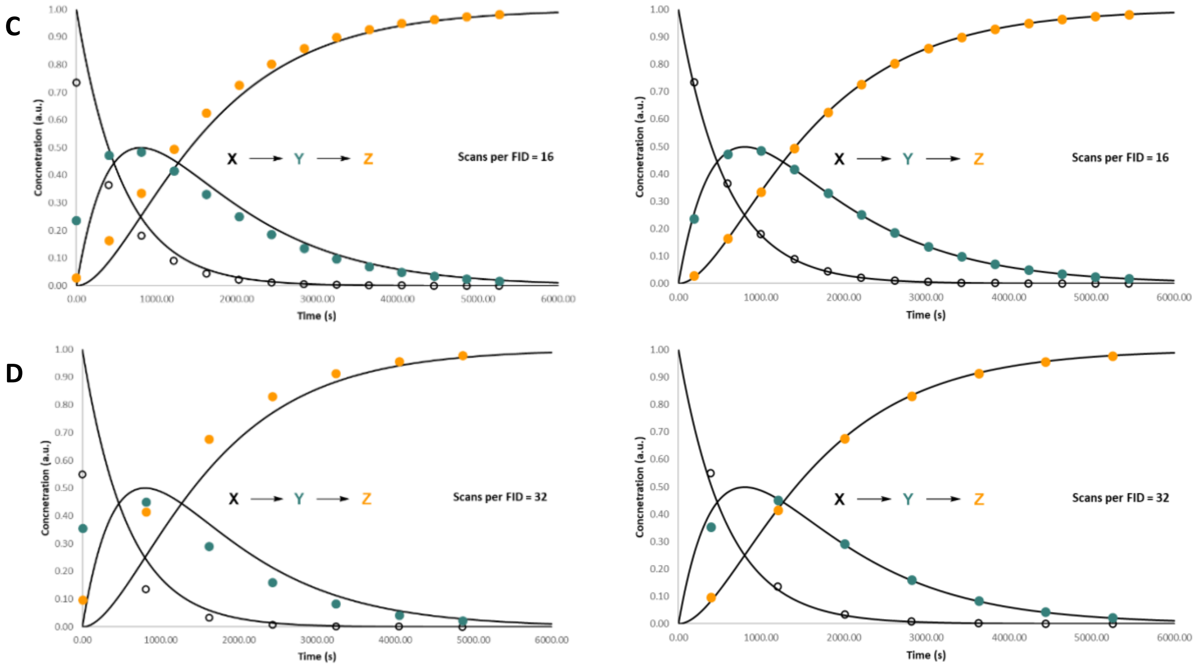

Contracting Average – Corrected Time

Moving Average – Corrected Time

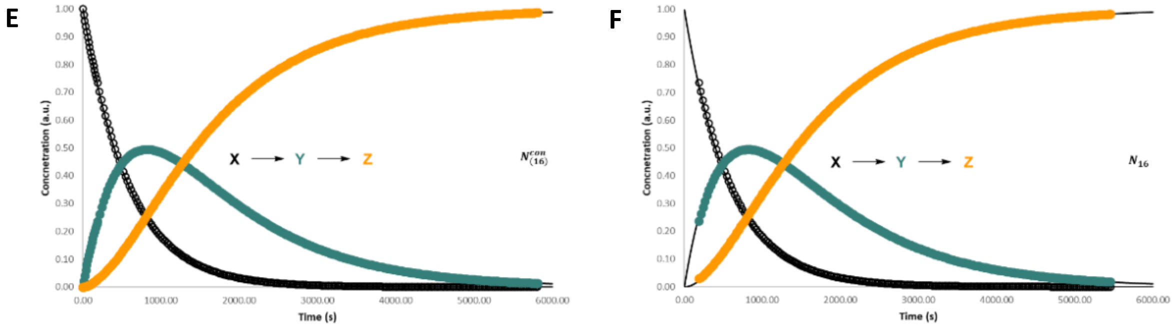

Figure S2. *Concentration-time simulated data: Monitoring for 6000 s with T_1^{max} = 5.1 s. A: Number of datapoints without averaging = 255. B: Signal-averaging with 8 scans per FID, equivalent to on-spectrometer averaging (number of datapoints = 28). C: Signal-averaging with 16 scans per FID, equivalent to on-spectrometer averaging (number of datapoints = 14). D: Signal-averaging with 32 scans per FID, equivalent to on-spectrometer averaging (number of datapoints = 7). For B-D, the time point for each datapoint is taken as the same as the first datapoint prior to averaging (left), and as the arithmetic mean (right). E: A contracting average ($N^{con}_{(16)} = 270$) is applied to the datapoints and the time points are corrected in the same manner as in C. F: A moving average ($N_{(16)} = 210$) is applied to the datapoints and the time points are corrected in the same manner as in C.*

S8. Pathway B: Reduction of τ_R and θ Increases Signal-to-Noise in Some Scenarios

As described by Equations 1, 2 and 3 (main text and reproduced below)

$$f_{max} = \sin\theta \qquad \text{Main Text, Equation 1}$$

$$\tau_R = -T_1^{max} \ln\left(\frac{1-\left(\frac{M_\tau}{M_0}\right)}{1-\cos\theta}\right) \qquad \text{Main Text, Equation 2}$$

$$\frac{S}{N} \propto (f_{max})\sqrt{scans\ per\ FID} \quad as\ \left(\frac{M_\tau}{M_0}\right) \to 1 \qquad \text{Main Text, Equation 3}$$

Signal-to-noise (S/N) can be plotted as a function of pulse angle (θ) for one scan per FID. When $\frac{M_\tau}{M_0} = 0.993$, S/N is maximised at θ = 89.596° (Section S8).

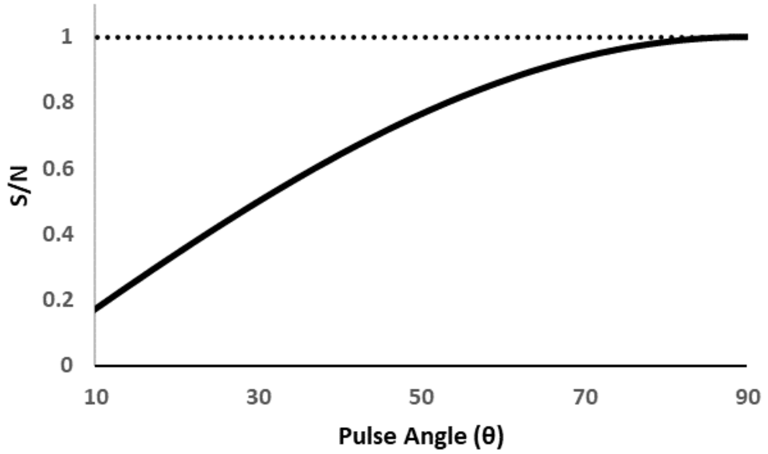

Figure S3: When scans per FID = 1, S/N increases as a function of the pulse angle.

For faster reactions, θ = 90° may not be desirable for reaction monitoring as the data density is reduced. To increase data density, a smaller θ can be implemented, which allows τ_R to be smaller whilst maintaining quantitation, Equation 2.

By reducing θ, τ_R can be decreased until the desired number of datapoints can be collected within the desired monitoring time (t_{Total}), Equation S5. However, the S/N of the single scan spectra is inherently lower when using θ < 90° (Equation S4). Similarly, reducing τ_R also increases the number of datapoints within a short time window (t_{Av}) over which signal-averaging may want to be applied.

$$\text{Number of datapoints over reaction} = \frac{t_{Total}}{\tau_R} \qquad \text{Equation S5}$$

$$\text{Number of datapoints over } \mathrm{t_{Av}} = \frac{t_{Av}}{\tau_R} + 1 \qquad \text{Equation S6}$$

When considering the number of spectra that can be recorded in this smaller time window, an interesting phenomenon is observed in which some values of $\theta < 90°$ can result in a higher S/N than when $\theta = 90°$. To illustrate this with an example, taking $t_{Av} = 24\ s$, $T_1^{max} = 5\ s$ and $\frac{M_\tau}{M_0} = 0.993$. The timelines for both processes are summarised in Figure S3.

When $\boldsymbol{\theta = 90°}$, using Equation 2 (main text)

$$\tau_R = -T_1^{max} \ln\left(\frac{1-\left(\frac{M_\tau}{M_0}\right)}{1-\cos\theta}\right)$$

$$\tau_R = 24.8\ s$$

Using Equation S6, the number of datapoints over $t_{Av} = 1.968$. As the number of datapoints must always be an integer, the number of datapoints over $t_{Av} = 1$.

∴ From Equation 3 (main text, reproduced below), the maximum S/N possible is:

$$\frac{S}{N} \propto (f_{max})\sqrt{scans\ pre\ FID} \qquad \text{Main Text, Equation 3}$$

$$\frac{S}{N} \propto (f_{max})\sqrt{1}$$

$$\frac{S}{N} \propto \sin\ (90)\sqrt{1}$$

$$\frac{S}{N} \propto 1.000$$

Repeating this logic for $\boldsymbol{\theta = 60°}$.

$$\tau_R = 21.3\ s$$

Number of datapoints over $t_{Av} = 2.006$, which rounds to 2.

$$\frac{S}{N} \propto (f_{max})\sqrt{2}$$

$$\frac{S}{N} \propto \sin\ (60)\sqrt{2}$$

$$\frac{S}{N} \propto 1.225$$

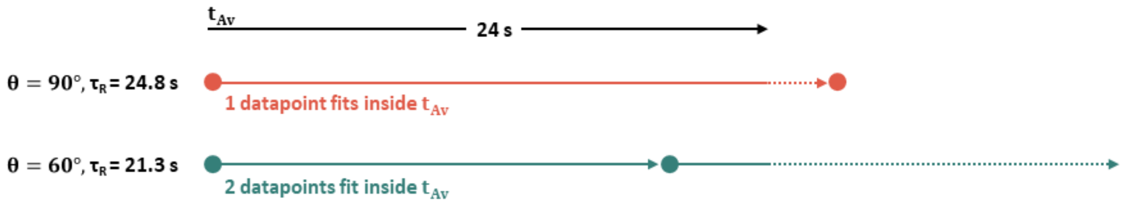

Figure S4: For $t_{Av} = 24\ s$, $T_1^{max} = 5\ s$ and $\frac{M_\tau}{M_0} = 0.993$. Using a 90° pulse allows only one spectrum to be acquired in the specified time window. Lowering the pulse angle to 60° reduces τ_R so that 2 datapoints can be acquired over the time window.

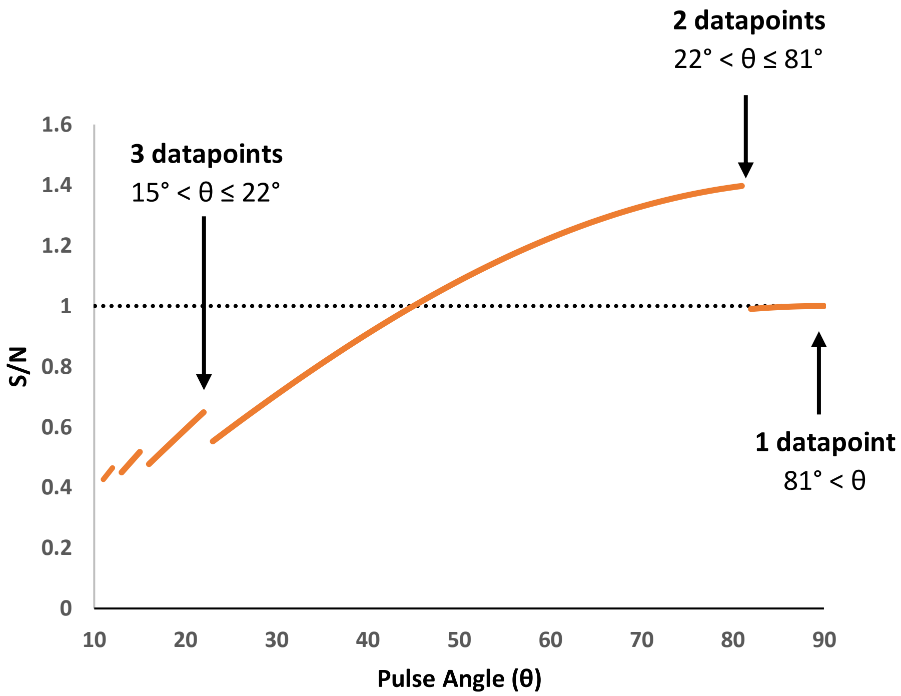

Figure S5: When scans per FID = is dependent on t_{Av}, S/N changes as a function of the pulse angle and the number of scans per FID. For $t_{Av} = 24\ s$, $T_1^{max} = 5\ s$ and $\frac{M_\tau}{M_0} = 0.993$, with the three largest disjunctions labelled. At θ = 45° (2 scans) and θ = 90° (1 scan) S/N = 1.

S9. Pulse Angles, the Ernst Equation and Quantitative Parameters

The Ernst Angle θ_E is that which gives the best S/N, given the T_1 and the repetition time τ, which is the sum of the acquisition time (AQ) and the delay between experiments (D1).

$$\cos(\theta) = \exp\left(-\frac{\tau}{T_1}\right) \quad where\ \tau = AQ + D1$$ Equation S7

The Ernst angle calculates the appropriate pulse angle to maximise sensitivity over a fixed time window, but only for qualitative NMR analysis. To modify the Ernst Equation so that experiments can be acquired in a quantitative manner τ must account for "full" relaxation $\left(\frac{M_\tau}{M_0}\right)$ of the magnetised nuclei back to magnetic equilibrium. τ_R is dependent on the pulse angle θ and the T_1 of the nuclei (Equation 2, main text and reproduced below).

$$\tau_R = -T_1^{max} \times \ln\left(\frac{1-\frac{M_\tau}{M_0}}{1-cos(\theta)}\right)$$ Equation 2

Full relaxation is described as a proportion of magnetisation at equilibrium, often given the value of 99.3% (which equates to ≅ 5×T_1 for full relaxation).

$$\frac{M_\tau}{M_0} = 0.993$$

Including the quantitation requirement into the Ernst Equation:

$$\cos(\theta) = \exp\left(-\frac{-T_1 \times ln\left(\frac{1-\frac{M_\tau}{M_0}}{1-cos(\theta)}\right)}{T_1}\right)$$ Equation S8

Simplifying to:

$$\cos(\theta) = \frac{1-\frac{M_\tau}{M_0}}{1-\cos(\theta)}$$ Equation S9

When $\frac{M_\tau}{M_0} = 0.993$, solving gives θ = 89.596°, or θ ≅ 90°. As such, to maximise signal in each single-scan spectrum, a 90° pulse should be used. Smaller pulse angles will result in lower signal when comparing signal in spectra acquired with the sample number of scans per FID, for this quantitation stipulation.

S10. Data Processing Validation

During post-acquisition processing, signal-averaging can be applied to the single scan FIDs, analogously to signal-averaging during acquisition. To demonstrate that the S/N enhancement from signal-averaging at time of acquisition is equivalent to signal-averaging during processing, a series of spectra were recorded in which the number of scans at the time of acquisition were varied.

Solution was prepared containing TFA (^{19}F singlet @ -75.06 ppm). All experiments were recorded consecutively without removing the sample from the probe. All parameters were identical and summarised in Table S14.

Table S14. *Signal-to-Noise comparison between signal-averaging during acquisition and during processing.*

Pulse Angle (θ)	90°
Receiver Gain	11.3
Acquisition Time	2.7 s
Delay between scans	20 s (excluding acquisition time)
Temperature	323 K

For the signal-to-noise calculation, the signal and noise regions were set at 0.2197 ppm wide and centred at -75.16 ppm and -74.43 ppm respectively. Spectra contained separately in the Supplementary Information

Table S15. *Signal-to-Noise comparison between signal-averaging during acquisition and during processing.*

Experiment	Number of Scans at acquisition	Scans summed during processing	S/N	Relative S/N	Theoretical S/N increase
1	1	N/A	1351.41	1.00	1
2	4	N/A	2701.29	2.00	2
3	8	N/A	3990.72	2.95	$2\sqrt{2}$ (2.83)
4	16	N/A	5609.80	4.15	4
6	1	4	2593.84	1.92	2
7	1	8	3787.93	2.80	$2\sqrt{2}$ (2.83)
8	1	16	5306.19	3.93	4

S11. - TopSpin 4.3.0 AU Programs for Averaging – With Annotations

The programs below have not been widely tested, use at your own risk. Both averaging AU programs are annotated here. Text files without annotations are contained separately in the Supplementary Information.

Two modifications of the averaging have been applied here. The moving average script describes averaging using a window of a fixed size (n). The contracting average script applies a growing window size at the beginning of the dataset until the desired averaging size (n) is reached, and a decreasing window size at the end of the dataset. Moving and contracting averaging are compared in Figure 3, main text.

Whilst both scripts can be used interchangeably, the moving average script should be preferentially applied to spectra acquired with phase cycling, as this will ensure all spectra have the correct number of scans for phase cycling. The rolling average script is appropriate for all other data.

S11.1. Averaging of FIDs

	Script	Comments
1.	**#include <inc/sysutil>**	Lines 1-5: Create variable names
2.	**int oexpno, newexpno, total_blocks;**	
3.	**char new_name[100], filedir[200], testdir[100], old_name[64];**	
4.	**#include <inc/getdataset>**	
5.		Line 6: Ask user for window size
6.	**GETINT("Number of scans per block?", i1)**	
7.	**oexpno = expno;**	
8.		Lines 7-12: Find number of consecutive 1D spectra in file
9.	**while (access(ACQUPATH("fid"), F_OK) == 0) {**	
10.	**expno++;**	
11.	**}**	
12.		
13.	**total_blocks = ((expno - oexpno) - (i1 - 1));**	Line 13: Calculate total number of spectra that will be created
14.	**(void) sprintf(new_name, "%s_%s_%d", name, "average", i1);**	
15.	**(void) sprintf(old_name, "%s", name);**	
16.		
17.	**TIMES(total_blocks)**	
18.		
19.	**expno = oexpno;**	
20.		Lines 17-40: Loop block – apply moving average.
21.	**DATASET(old_name, expno, procno, disk, user)**	
22.		
23.	**WRPA(new_name, expno, procno, disk, user)**	
24.	**DATASET(new_name, expno, procno, disk, user)**	Line 19-23: Copy current spectrum to a folder called *new_name*.
25.	**DATASET2(new_name, expno, procno, disk, user)**	
26.		
27.	**STOREPAR("DC", 1.0)**	
28.	**STOREPAR("TI", "result of fidadd")**	
29.	**STOREPARS("TI", "result of fidadd")**	
30.		Line 27-29: Store parameter values.
31.	**TIMES2(i1-1)**	
32.	**oexpno = oexpno+1;**	
33.		
34.	**DATASET3(old_name, oexpno, procno, disk, user)**	Lines 31-37: Add sequential FIDs to the FID in the folder *new_name* from the folder *old_name*.
35.	**ADDFID**	
36.		
37.	**END**	
38.	**oexpno = expno+1;**	

39.		
40.	END	
41.		Line 38: Repeat for the next experiment number.
42.	// Delete all processed data in newly created files.	
43.		
44.	GETCURDATA	
45.	oexpno = expno + i1;	Lines 42-53: Delete all *processed* data (copied from the original data) in the new folder.
46.	expno = - total_blocks - (i1-1) + oexpno;	
47.		
48.	TIMES(oexpno-expno)	
49.	(void) sprintf(filedir, "%s/%s/%d/pdata/%d", disk, new_name, expno++, procno);	
50.	(void) unlinkpr (filedir);	
51.	END	
52.		
53.	QUITMSG(filedir);	

S11.2. Averaging of FIDs with Additional Contracting

	Script	Comments
1.	#include <inc/sysutil>	Lines 1-5: Create variable names
2.	int oexpno, newexpno, total_blocks, counter=1, back_counter, block_size, date, start_exp_num, arbnum;	
3.	char new_name[100], filedir[200], testdir[100], old_name[64], arb_title[64], test1[200], test2[200];	
4.	double new_scan_num;	
5.	#include <inc/getdataset>	
6.		Line 7: Ask user for window size
7.	GETINT("Number of scans per block?", i1)	
8.	oexpno = expno;	
9.	start_exp_num = expno;	Lines 8-14: Find number of consecutive 1D spectra in file
10.		
11.	while (access(ACQUPATH("fid"), F_OK) == 0) {	
12.	expno++;	
13.	}	
14.		Line 15: Calculate total number of spectra that will be created
15.	total_blocks = (expno - oexpno);	
16.		
17.	(void) sprintf(new_name, "%s_%s_%d", name, "average_roll_both", i1);	
18.	(void) sprintf(old_name, "%s", name);	
19.		Lines 20-53: Loop block – apply averaging.
20.	TIMES(total_blocks)	
21.		
22.	expno = oexpno;	
23.	DATASET(old_name, expno, procno, disk, user)	Line 22-26: Copy current spectrum to a folder called *new_name*.
24.	WRPA(new_name, expno, procno, disk, user)	
25.	DATASET(new_name, expno, procno, disk, user)	
26.	DATASET2(new_name, expno, procno, disk, user)	
27.		
28.	STOREPAR("DC", 1.0)	Line 28: Store parameter values.
29.		
30.	if (counter < i1) {	
31.	block_size = counter;	Lines 30-32: Set up block size for the
32.	}	
33.	else {	

```
34.             block_size = i1;
35.                     }
36.             new_scan_num = expno;
37.             TIMES2(block_size-1)
38.                     oexpno = oexpno-1;
39.                     new_scan_num = new_scan_num + oexpno;

40.                     DATASET3(old_name, oexpno, procno, disk, user)
41.             ADDFID
42.
43.             END
44.
45.             new_scan_num = (new_scan_num / block_size) - start_exp_num
    + 1;
46.             (void) sprintf(arb_title, "%s %.2f", "scan number",
    new_scan_num);
47.             STOREPAR("TI", arb_title)
48.             STOREPARS("TI", arb_title)
49.
50.             oexpno = expno+1;
51.             counter++;
52.
53.     END
54.
55.     back_counter = i1 - 1;
56.     counter = 1;
57.
58.     // the backwards roll
59.     TIMES(i1 - 1)
60.             expno = total_blocks + start_exp_num - 1;
61.             oexpno = expno;
62.             DATASET(old_name, expno, procno, disk, user)
63.             arbnum = expno + counter;
64.             WRPA(new_name, arbnum, procno, disk, user)
65.             DATASET(new_name, arbnum, procno, disk, user)


66.             DATASET2(new_name, arbnum, procno, disk, user)
67.
68.             STOREPAR("DC", 1.0)
69.
70.             block_size = back_counter;
71.
72.             new_scan_num = total_blocks + start_exp_num - 1;
73.             TIMES2(block_size-1)
74.                     oexpno = oexpno-1;
75.                     new_scan_num = new_scan_num + oexpno;

76.                     DATASET3(old_name, oexpno, procno, disk, user)
77.             ADDFID
78.
79.             END
80.
81.             new_scan_num = (new_scan_num / block_size) - start_exp_num
    + 1;
82.             (void) sprintf(arb_title, "%s %.2f", "scan number",
    new_scan_num);
```

growing window size for initial spectra. Lines 33-35: Set up block size for standard moving average.

Lines 37-41 Add sequential FIDs to the FID in the folder *new_name* from the folder *old_name*.

Lines 45-48: Calculate the scan number and save it as 'TI' in TopSpin *Note: this value acts as a reference only.*

Line 50: Repeat for the next experiment number.

Lines 55-90: Repeat of lines 20-53 but for the decreasing window size for final spectra.

```
83.             STOREPAR("TI", arb_title)

84.             STOREPARS("TI", arb_title)
85.
86.             oexpno = expno+1;
87.             back_counter--;
88.             counter++;
89.
90. END
91.
92. // Delete all processed data in newly created files.
93.
94. GETCURDATA
95. oexpno = expno + i1;
96. expno = - total_blocks - (i1-1) + oexpno;
97.
98. TIMES(oexpno-expno)
99. (void) sprintf(filedir, "%s/%s/%d/pdata/%d", disk, new_name, expno++, procno);
100.(void) unlinkpr (filedir);
101.END
102.
103.QUITMSG(filedir);
```

Lines 92-103: Delete all *processed* data (copied from the original data) in the new folder.

References

S1 Wei, R.; Dickson, C. L.; Uhrín, D.; Lloyd-Jones, G. C. Rapid Estimation of T1 for Quantitative NMR. *J. Org. Chem.* **2021**, *86*, 9023–9029.

S2 See Figure 1 in: Ben-Tal, Y.; Boaler, P. J.; Dale, H. J. A.; Dooley, R. E.; Fohn, N. A.; Gao, Y.; García-Domínguez, A.; Grant, K. M.; Hall, A. M. R.; Hayes, H. L. D.; Kucharski, M. M.; Wei, R.; Lloyd-Jones, G. C. Mechanistic Analysis by NMR Spectroscopy: A Users Guide. *Prog. Nucl. Magn. Reson. Spectrosc.* **2022**, *129*, 28–106.

S3 Dale, H. J. A.; Hodges, G. R.; Lloyd-Jones, G. C. Kinetics and Mechanism of Azole N−π*-Catalyzed Amine Acylation. *J. Am. Chem. Soc.* **2023**, *145*, 18126–18140.
